# Personality in the chimpanzees of Gombe National Park

**DOI:** 10.1038/sdata.2017.146

**Published:** 2017-10-24

**Authors:** Alexander Weiss, Michael L. Wilson, D. Anthony Collins, Deus Mjungu, Shadrack Kamenya, Steffen Foerster, Anne E. Pusey

**Affiliations:** 1 National Evolutionary Synthesis Center, Durham, NC 27705, USA; 2 Department of Psychology, School of Philosophy, Psychology and Language Sciences, The University of Edinburgh, Edinburgh, EH8 9JZ, UK; 3 Scottish Primate Research Group, UK; 4 Department of Anthropology, University of Minnesota, Minneapolis, MN 55455, USA; 5 Department of Ecology, Evolution and Behavior, University of Minnesota, St Paul, MN 55108, USA; 6 Institute on the Environment, University of Minnesota, St Paul, MN 55108, USA; 7 Gombe Stream Research Centre, The Jane Goodall Institute, P.O. Box 185, Kigoma, Tanzania; 8 Evolutionary Anthropology, Duke University, Durham, NC 27708, USA

**Keywords:** Biological anthropology, Psychology

## Abstract

Researchers increasingly view animal personality traits as products of natural selection. We present data that describe the personalities of 128 eastern chimpanzees (*Pan troglodytes schweinfurthii*) currently living in or who lived their lives in the Kasekela and Mitumba communities of Gombe National Park, Tanzania. We obtained ratings on 24 items from an established, reliable, well-validated questionnaire used to study personality in captive chimpanzee populations. Ratings were made by former and present Tanzanian field assistants who followed individual chimpanzees for years and collected detailed behavioral observations. Interrater reliabilities across items ranged from acceptable to good, but the personality dimensions they formed were not as interpretable as those from captive samples. However, the personality dimensions corresponded to ratings of 24 Kasekela chimpanzees on a different questionnaire in 1973 that assessed some similar traits. These correlations established the repeatability and construct validity of the present ratings, indicating that the present data can facilitate historical and prospective studies that will lead to better understanding of the evolution of personality in chimpanzees and other primates.

## Background & Summary

Personality variation exists in diverse taxa, including insects, fish, reptiles, birds, and mammals^1^. Studies of nonhuman primates often involve long-term behavioral observations on recognized individuals^2–4^ and are therefore well-suited for studies of personality. Unfortunately, our understanding of personality variation and its evolutionary significance among primates is limited, because only a small number of species have been studied in the wild^4–19^.

Jane Goodall drew the attention of a global audience with vivid depictions of the personalities of eastern chimpanzees (*Pan troglodytes schweinfurthii*) at Gombe National Park^20,21^, yet only one attempt has been made to quantify these personality traits systematically. This study involved ratings of 24 chimpanzees from the Kasekela community on a personality questionnaire, the Emotions Profile Index (EPI), made in 1973 by researchers (students and post-docs) who had been studying these chimpanzees and who had known them for several months to several years^7^. The EPI ratings were consistent across raters. They indicated that females were more Trustful, Timid, and Depressed, but less Distrustful and Gregarious than males, and that higher ranking males were more Aggressive and less Timid while lower ranking males were more Dyscontrolled and more Timid^7^. Furthermore, the personality profile of one female (Passion) differed markedly from other females: she was more Aggressive, Depressed, and Distrustful, and less Trustful, Timid, Controlled, and Gregarious. Strikingly, starting in 1975, Passion and her daughter Pom killed and ate at least four infants of other females in the community^8^.

Chimpanzees are among our closest living relatives, sharing a common ancestor with humans a mere six to eight million years ago^22,23^. Studies of chimpanzees have illuminated many aspects of human evolution, including tool use^24^ and other cultural traits^25^, hunting^26^, and intergroup violence^27^. Long-term studies of wild chimpanzees can be a rich source of insight into the evolution of human personality, and behavior more generally. The long-term study at Gombe has collected systematic data on the behavior of individual chimpanzees for decades. These data provide ample evidence of individual differences in behavioral changes at adolescence^28^, female fertility and phenotypic quality^29^, rate of learning tool techniques^30^, female dominance rank and range use^31^, male reproductive strategies^32^, and male participation in hunting and territorial behavior^33^. However, because the data collection methods at Gombe National Park were not designed to capture personality traits, work is needed to produce data that can be directly compared with other personality studies.

Our aim is to lay the groundwork for the systematic study of personality in wild chimpanzees. As a first step towards such a study, we collected personality trait ratings of the Gombe chimpanzees made by experienced, long-term observers. These ratings are continuous variables that indicate, for each chimpanzee, where they stand in comparison to other chimpanzees on a trait. We obtained ratings on 128 chimpanzees including individuals that were observed in detail while alive but who have since died. The present data therefore resemble ratings of the personalities of living and deceased historical figures by people who knew the individuals well, or by historians^34^.

In this paper, we describe the personality ratings data that we collected, provide background information on how this was done, and describe the characteristics of the chimpanzees and their raters. In addition, we address three questions about the data that will be useful for future studies: (a) to what degree do raters agree in their ratings of individual chimpanzees? (b) what personality dimensions do factor analyses and principal components analyses reveal as underlying the correlations among personality traits? (c) for a subset of chimpanzees, to what extent do the ratings obtained in this study agree with the ratings on the EPI made in 1973 by different raters?^7^

Examining correlations between the ratings that we gathered and the EPI ratings enables us to assess the ratings’ construct validity. Evidence for construct validity consists of high correlations between dimensions on independent measures that represent similar psychological constructs and low to negligible correlations between dimensions on independent measures that represent different psychological constructs^35,36^. For example, independent measures of sociability or vigilance should be correlated, but sociability measures should not be correlated with vigilance measures. Finally, because the EPI ratings were made nearly forty years earlier, these analyses indicate the extent to which these personality traits are stable over decades.

## Methods

### Site and communities

**Figure 1:**
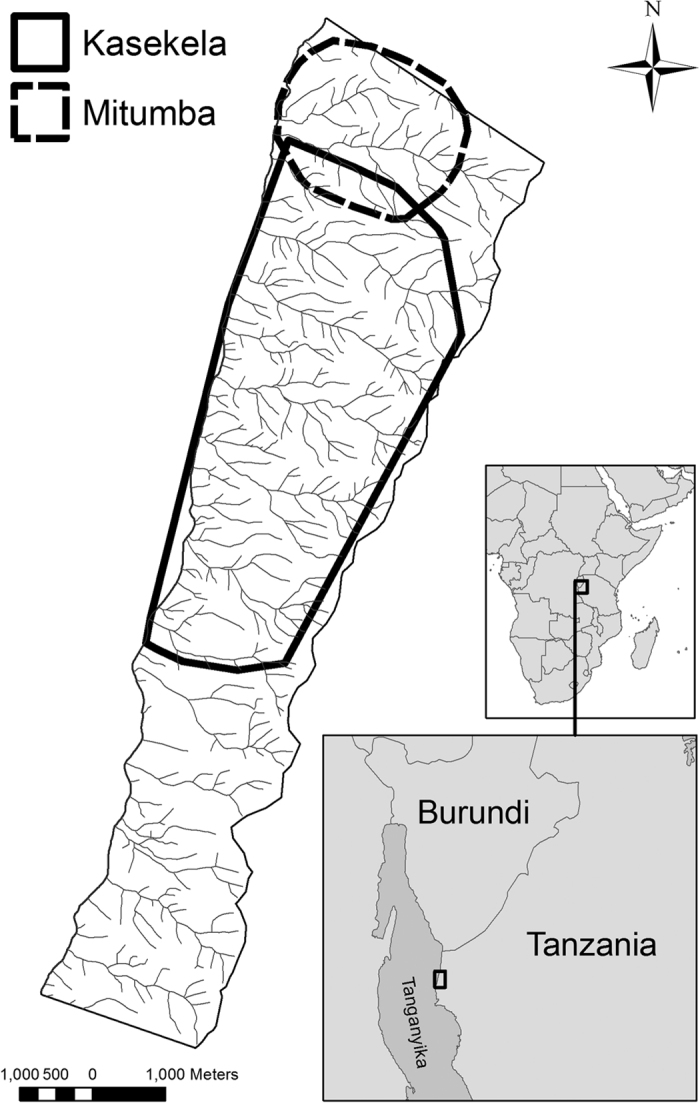
Map of Gombe National Park. The ranges of the Kaskela and Mitumba communities cover the years 2000 to 2009. Earlier ranges are described in Williams *et al.*^82^ and Wilson *et al.*^83^

Gombe National Park consists of a narrow, 35 km^2^ strip of mountainous terrain along the eastern edge of Lake Tanganyika in Tanzania. It contains a complex mosaic of habitat transitions from riverine forest in the valleys to deciduous woodland and grassland on the ridges^37^. Gombe contains three communities of chimpanzees (from north to south): Mitumba, Kasekela and Kalande (Fig. 1). Goodall began habituating the Kasekela community to the presence of human researchers in 1960 (ref. 3), and demographic records have been continuously kept ever since. The chimpanzees were provisioned with bananas at an artificial feeding station from 1963 to 2000, with daily records made of their behavior^3,38^. Since the early 1970s, Tanzanian field assistants have conducted almost-daily dawn-to-dusk focal follows^39^ of chimpanzees as they travel throughout their range. During these focal follows, the field assistants systematically record changes in party composition and location and make a narrative record of the behavior of the focal chimpanzee and events in the group^3,38^. In each focal follow, the field assistants focused attention on a single individual, observing all of its behavior: feeding, fighting, mating, parenting, grooming, tool use, hunting, and so forth. Focal targets varied daily, rotating through most of the chimpanzees in each community each month, providing the field assistants opportunities to get to know many chimpanzees in great detail. Between 1966 and 2014 the Kasekela community varied in size from 38 to 63 individuals. The Kahama community consisted of at least 15 individuals (and possibly some additional unhabituated females) that split from the Kasekela community in the early 1970s. Kasekela chimpanzees killed at least six individuals from Kahama in a series of violent attacks, and the Kahama community ceased to exist by 1977 (ref. 3).

Efforts to habituate the Mitumba community started in 1985. Field assistants regularly provisioned Mitumba chimpanzees at an artificial feeding area from 1992–2000, and collected demographic and behavioral data. From the mid-1990s, field assistants conducted focal follows using methods identical to those used for the Kasekela community. Almost all members of the Mitumba community were habituated by 1994 (ref. 40). Between 1994 and 2014 the community varied in size from 20 to 28 individuals.

### Chimpanzees

We initially collected 494 personality ratings that described 141 eastern chimpanzees who lived or were living in Gombe National Park from ca. 1963 to the present. We excluded ratings of chimpanzees that were likely to be less informative or valid. The remaining data consisted of chimpanzees who (a) survived to be at least five years old, (b) had been observed for at least two years, (c) had been sighted on at least 100 days over the qualifying period, i.e., when that chimpanzee was a regular member of the community, and, because the first field assistants were hired in late 1969 and the early to mid-1970s, (d) had been alive and observed after 1970. The excluded chimpanzees included one chimpanzee from Mitumba who did not survive to be at least five years old (criterion **a**) and nine chimpanzees from Kasekela who had not been sighted on at least 100 days (criterion **c**). In addition, a later inspection of records after data had been collected revealed that three chimpanzees from Kasekela who had been rated may have been confused with other chimpanzees, and so we excluded ratings of these chimpanzees as well.

After excluding the 34 ratings of these 13 individuals, the data set consisted of 460 personality ratings of 128 chimpanzees. This data set made up our working sample and, unless noted, will be described throughout. This sample comprised 56 males and 72 females who either belonged to the Kasekela community (46 males and 58 females including 6 males and 6 females that fissioned into the Kahama community), the Mitumba community (10 males and 11 females), or who had been members of both communities (3 females). Sixty-three of these individuals (32 males and 31 females) were alive on 11 October 2010, the date we began to collect ratings.

### Ratings

#### Questionnaire

Personality ratings were made on a modified form of the Hominoid Personality Questionnaire (HPQ)^41^. A full history of the development of the HPQ is available elsewhere^42^; a brief history follows. The original HPQ consists of 54 items and was an expanded version of the 48-item Orangutan Personality Questionnaire^43^, which was, in turn, an expanded version of the 43-item Chimpanzee Personality Questionnaire^44^. Of the 43 Chimpanzee Personality Questionnaire items, 41 were sampled from a taxonomy of the five human personality dimensions^45^. The two additional items, ‘clumsy’ and ‘autistic,’ were devised by King and Figueredo, the authors of the Chimpanzee Personality Questionnaire^44^. Of the additional items in the full HPQ, five were chosen to increase the number of traits related to Openness, three were chosen to increase the number of traits related to Neuroticism, and three were chosen to increase the number of items related to Conscientiousness.

As noted in papers describing the development of the HPQ^41,43,44^, only items considered applicable to chimpanzees and other nonhuman primates were sampled. Each HPQ item comprises a trait adjective paired with one to three sentences that clarified the trait adjective’s meaning in the context of chimpanzee behavior. For example, the clarifying sentence for the item ‘helpful’ was ‘Subject is willing to assist, accommodate, or cooperate with other chimpanzees.’ The HPQ also includes printed instructions that asked raters to rate each item on a scale ranging from 1 (‘Displays either total absence or negligible amounts of the trait’) to 7 (‘Displays extremely large amounts of the trait’), to base judgments on their understanding of typical chimpanzee behavior, and not to discuss their ratings with others.

Two important things to note about the HPQ are: (a) although the items included representatives of traits associated with the well-established five human personality dimensions—Neuroticism, Extraversion, Openness to Experience, Agreeableness, and Conscientiousness—the questions were adapted for use in nonhuman primates; and (b) traits across the five human dimensions were chosen not to try and impose a human personality structure onto chimpanzee or primates, but to ensure that a wide range of traits related to behavior, affect, and cognition have been sampled^41,43,44^. Therefore, although these items may be similar in humans and other primates, they are not identical, and ratings of primates on this questionnaire do not necessarily result in dimensions corresponding to human personality dimensions. For example, a study of rhesus macaques found six personality dimensions—Confidence, Openness, Dominance, Friendliness, Activity, and Anxiety^10^—three of which resembled those found in earlier studies of this species, even though those earlier studies used different instruments to assess personality^46^.

The HPQ and its predecessors have predominated in research on the personalities of chimpanzees and the other great apes^18,41,43,44,47,48^. The Chimpanzee Personality Questionnaire was used to assess the personalities of 100 zoo-housed chimpanzees, and ratings revealed the presence of five personality dimensions—Extraversion, Conscientiousness, Agreeableness, Neuroticism, and Openness—similar to those found in humans, and an additional chimpanzee-specific dimension labeled ‘Dominance’^44^. The interrater reliabilities of these dimensions were on par with interrater reliabilities derived in studies of humans including self- and spouse-ratings and studies in which individuals were rated by peers^44^. Later studies of chimpanzees using the Chimpanzee Personality Questionnaire or the HPQ demonstrated that these ratings are repeatable^49^, associated with behavior^50^, and linked to genetic variation^51–53^, and neurobiology^54^. Moreover, although the HPQ originated in zoo research and in an English-speaking country, the HPQ and related instruments have been used successfully for measuring chimpanzee personality in other contexts and/or in other languages. Studies of chimpanzees in a naturalistic sanctuary in the Republic of Congo using the English-language version and a French translation of the Chimpanzee Personality Questionnaire^55^; Yerkes National Primate Research Center using the English language version of the Chimpanzee Personality Questionnaire^47^; and zoos, a sanctuary, and two research institutes in Japan using a Japanese translation of the HPQ^41^ all revealed personality dimensions similar to those identified in King and Figueredo’s original study of zoo chimpanzees. The interrater reliabilities observed in these studies were also consistent with those found in King and Figueredo’s original study.

For the present study, we made three modifications to the HPQ. The first modification was to change references to zoo-specific environmental features in clarifying sentences, e.g., ‘the enclosure,’ to neutral phrases, e.g., ‘the environment’. The second modification was to translate the HPQ items into Swahili, the national language of Tanzania (most raters were native speakers of the local language, Kiha, but all were fluent in Swahili, the working language at Gombe). The translation was done by Munira Massoud, a Tanzanian student at the University of Minnesota, fluent in both Swahili and English. The translations were then cross-checked by specialists in chimpanzee research who were competent in both English and Swahili (SK and MLW). In addition, we noticed that two items (‘innovative’ and ‘inventive’) had the same adjective label in Swahili (‘mbunifu’), although the behavioral descriptors differed. With the help a co-author (DAC) who was fluent in English and Swahili, we changed the adjective label to ‘mtengenezaji’. Because the correlation between the items ‘innovative’ and ‘inventive’ was higher in the Mitumba sample (*r*=0.53, *P*=0.014), where they had different adjective labels, than in the Kaskela sample (*r*=0.22, *P*=0.022), there is no evidence that the responses on these items by raters of the Kasekela chimpanzees were biased because these items had the same label. The third modification was to reduce the length of the questionnaire to 24 items. Although reducing the number of items risked reducing the reliability of scales^56^, we considered this risk acceptable, because we considered it more important to obtain ratings on as many chimpanzees as possible, and we were concerned that including too many items would make the raters’ response burdens excessive. In fact, collecting data on this sample using the 24-item version of the questionnaire took nearly two months.

When choosing items to include in the shorter version of the Swahili-language HPQ, we sought to meet four criteria. The first was that for each of the six dimensions identified in previous studies^41,44^ we should, if possible, have four items, and wherever possible, have an even number of items that were positively and negatively associated with those dimensions. The second was to maximize the item overlap with studies that have used earlier versions of the questionnaire, e.g., the Chimpanzee Personality Questionnaire. To these ends we decided that each dimension should be represented by at most 1 of the 11 items that were in the HPQ, but that were not in the Chimpanzee Personality Questionnaire. We met these first two criteria for Dominance, Extraversion, Conscientiousness, Agreeableness, and Neuroticism. However, for Openness, we had to select two Openness items from the HPQ, because only two of the Chimpanzee Personality Questionnaire items were related to Openness. Our third criterion was that the items that we chose had displayed high interrater reliabilities in previous studies that used the Chimpanzee Personality Questionnaire or HPQ to rate chimpanzees. The fourth criterion was to choose items that were strongly related to the dimensions, i.e., had high absolute loadings on these dimensions in previous studies.

The full length English language version of the Hominoid Personality Questionnaire for chimpanzees is provided in Supplementary File 1, the full-length Swahili version in Supplementary File 2, and the brief, 24-item Swahili version in Supplementary File 3.

#### Raters

This study benefits from the experience of an extraordinary group of individuals, the Tanzanian field assistants at Gombe. These men spent up to 35 years observing chimpanzees in the wild, which is more time than anyone else in history with the possible exception of local field assistants working at other long-term studies such as Mahale^57^. Chimpanzees can live well into their 50s in the wild, but at Gombe, most die before they are 40 (ref. 58). The most experienced field assistants thus observed many chimpanzees for their entire lives, from birth to death. These field assistants have limited formal education, with little or no understanding of English, the language in which most of the technical scientific literature is written. Nonetheless, nobody knows these individual chimpanzees better than they do.

Eighteen of these field assistants who had extensive daily experience in observing and tracking the chimpanzees and recording their behavior^3,38^ rated the chimpanzees. Each field assistant rated between 21 and 43 chimpanzees (mean subjects per rater=27.4, *s.d.*=5.6). One field assistant accidentally rated one chimpanzee a second time three days later; in this case, the correlation between the ratings across the 24 items was 0.66. Each chimpanzee was rated by two to nine field assistants (mean raters per subject=3.5, *s.d.*=1.3).

Of these 18 field assistants, 10 were still working with the chimpanzee community that they rated, two had traded work stations (Q moved to Kasekela and O moved to Mitumba), and so were asked to rate the chimpanzees from the community where they had worked longest (Mitumba for Q, and Kasekela for O), four were retired, and two were no longer employed. The 12 field assistants who were still employed had been working for a mean of 16.7 years at the time they rated the chimpanzees and knew the chimpanzees they rated for 1 to 25 years (mean=10.7, *s.d.*=5.3). The six field assistants who had retired or who were no longer employed had worked for a mean of 28.3 years at the time they rated the chimpanzees and had known the chimpanzees they rated for less than one year to 35 years (mean=11.4, *s.d.*=7.7).

The starting dates of employment ranged from 1969 to 2003 (median=1987.5). The eight field assistants who were no longer working with the chimpanzee community that they rated ended their work with that community from one to five years before doing the ratings (median=4.5). This meant that in many cases, ratings of the chimpanzees by different raters reflect the same chimpanzee over different parts of that chimpanzee’s life. Therefore, instead of providing a single age for each chimpanzee in this dataset, we determined, for each chimpanzee-rater pairing, the median age of the chimpanzee. For further details on which chimpanzees were rated by which field assistants and the employment dates of field assistants for the Kasekela and Mitumba communities, see Supplementary Tables 1 and 2, respectively.

#### Procedure

Ratings were conducted when field assistants were not watching or interacting with chimpanzees. Most ratings of the Kasekela chimpanzees were conducted at the Jane Goodall Institute’s Education Centre in Kigoma, Tanzania. Up to two field assistants completed questionnaires at any given time while sitting at separate desks on opposite sides of a room. A few field assistants completed some of their questionnaires at home. Most ratings of the Mitumba chimpanzees were conducted in Gombe National Park, either in a room with two desks or at the field assistant’s home. During ratings of the Kasekela chimpanzees and the Mitumba chimpanzees, an interpreter was on hand to answer questions about the questionnaire or about a specific item or set of items. Most of the field assistants completed the questionnaires on their own. However, in a few cases, mostly confined to some of the older, retired field assistants, the interpreter read each question to the field assistant and recorded his answer.

We covered the field assistants’ travel expenses, meals, and, when necessary, their accommodation costs. In addition, field assistants who were still employed were paid 2,000 Tanzanian shillings (~$1.38 in 2010 and ~$1.26 in 2011) for each chimpanzee they rated and retired field assistants and field assistants who were no longer employed were paid 4,000 Tanzanian shillings (~$2.78 in 2010 and ~$2.52 in 2011) for each chimpanzee they rated. Thus, for each chimpanzee rated, field assistants who were still employed earned ~3.6 times the median net hourly wage in 2012 in Tanzania (550 Tanzanian shillings)^59^; and field assistants who were retired or no longer working earned ~7.3 times the median hourly wage.

#### Missing data

Of the 11,040 possible responses (24 items for 460 ratings), 32 were missing. The missing responses came from ratings of 23 chimpanzees by 11 field assistants. In 21 cases (the rating of one chimpanzee by one rater) one item was not completed, in three cases two items were not completed, and in one case, five items were not completed. For the purposes of our analyses, as in similar studies of wild nonhuman primates^4,10,18^, we substituted missing item data with the mean for that item.

#### Data aggregation

For the purposes of analyses other than obtaining interrater reliabilities, including data reduction and examining correlations between these ratings and the earlier ratings on the EPI^7^, we calculated the mean personality rating across raters for each personality variable for each chimpanzee.

#### Factor scores

Based on the results of previous studies^41,44^ and using items that we found to be reliable (see the ‘Interrater reliabilities of items’ section in Technical Validation), we created six personality dimension variables to represent each of the six chimpanzee personality dimensions. We created these scores for the raw ratings (the non-aggregated data) and for the ratings that had been averaged across raters (the aggregated data). In both the non-aggregated and aggregated data, missing ratings were substituted with the mean of that rating.

## Data Records

Trait ratings and demographic details for the 128 chimpanzees are available in two comma-separated (.csv) text files stored in the Open Science Framework (Data Citation 1). The file gombe_460.csv consists of 460 records representing two to nine ratings for each of the 128 chimpanzees. The variables include a three to four alphanumeric code indicating the chimpanzee’s anonymized ID (chimpcode), a single letter to identify the field assistant (ratercode), a variable (sex) indicating whether the chimpanzee was male (1) or female (0), and a variable (community) indicating whether the field assistant knew the chimpanzee while the chimpanzee was a member of the Kasekela (1) or Mitumba (0) community. This file also includes variables indicating the chimpanzee’s year of birth (chimp_YOB), the variables (month), (day), and (year) indicating the date when the field assistant rated the chimpanzee, four digit variables representing the year the field assistant started (rater_start_year) and the year the field assistant either stopped (rater_end_year) working at Gombe or the year that they performed the ratings, whichever was earlier, and two variables that indicated the year that the chimpanzee was first (chimp_start_year) and last (chimp_end_year) seen (and/or was regularly observed) in that community. We used (rater_start_year) and (rater_end_year) and (chimp_start_year) and (chimp_end_year) to create variables that estimated the year in which each field assistant first saw the chimpanzee (first_expr) and last saw the chimpanzee (last_expr). These two variables and a variable indicating their median (hpq_midpoint_yr) are also included in the file. Then, using (first_expr), (last_expr), and (hpq_midpoint_yr), and (chimp_YOB), we estimated the age of the chimpanzee when he or she was first observed by the field assistant (hpq_age_firstexpr), last observed by the field assistant (hpq_age_lastexpr), and the median of these values (hpq_midpoint_age).

Finally, this file includes variables indicating ratings on the 24 personality traits and variables representing the raw scores on the six personality dimensions identified and described in previous studies^41,44^. There were 48 variables representing the 24 personality traits: dominant (dom), excitable (exct), helpful (help), sensitive (sens), sociable (soc), reckless (reckl), sympathetic (symp), inquisitive (inqs), active (actv), innovative (innov), inventive (invt), impulsive (impl), curious (cur), solitary (sol), dependent/follower (depd), stable (stbl), predictable (pred), decisive (decs), cool (cool), vulnerable (vuln), individualistic (indv), thoughtless (thotl), conventional (conv), and unemotional (unem). The first 24 personality trait score variables had the suffix ‘.raw’, e.g., (indv.raw), to indicate that missing ratings data for these variables were coded ‘NA’. The second 24 personality trait score variables had the suffix ‘.meansub’, e.g., (indv.meansub), to indicate that missing ratings data for these variables were substituted with the mean for that trait. Both sets of personality trait score variables ranged from 1 to 7. The personality dimension raw scores generated from the 19 reliable items were Dominance (dominance), Extraversion (extraversion), Conscientiousness (conscientiousness), Agreeableness (agreeableness), Neuroticism (neuroticism), and Openness (openness). We computed these scores and scaled them so that they ranged from 1 to 7 using the formulae:

dominance=(dom.meansub-depd.meansub+decs.meansub+8)/3

extraversion=(−sol.meansub+soc.meansub-indv.meansub+actv.meansub+16)/4

conscientiousness=(−impl.meansub-reckl.meansub+pred.meansub+16)/3

agreeableness=(symp.meansub+help.meansub+sens.meansub)/3

neuroticism=(−stbl.meansub+exct.meansub+8)/2

openness=(invt.meansub+inqs.meansub+cur.meansub+innov.meansub)/4

The file gombe_128.csv consists of 128 records, each representing a single chimpanzee. Each record in this file represents the mean score across the two to nine field assistants who rated that chimpanzee. The variables include the two to three letter code indicating the chimpanzee’s anonymized ID (chimpcode), and a variable (sex) indicating whether the chimpanzee was male (1) or female (0). The variable (kasekela) indicated the proportion of raters who observed that chimpanzee as a member of the Kasekela community. For example, P70 was assigned a 1 on this variable because he was rated by three field assistants who observed him as a member of the Kasekela community, G74 was assigned a 0 on this variable because he was rated by six field assistants who observed him as a member of the Mitumba community, and E131 was assigned a score equal to 1/7 because she was rated by one field assistant who observed her as a member of the Kaskela community and six who observed her as a member of the Mitumba community. The file gombe_128.csv also includes 24 variables representing the 24 personality trait ratings described above. These values for each chimpanzee were obtained by computing the mean of the items across the raters and were based on the scores in gombe_460.csv where missing values were substituted by the mean, e.g., (dom.meansub). Each of these variables ranges between 1 and 7. This data set also includes six personality dimension raw scores: (dominance), (extraversion), (conscientiousness), (agreeableness), (neuroticism), and (openness). These variables were computed using the formulae above, but were based on the aggregated trait scores in this data file. These variables representing the six personality dimensions thus also ranged from 1 to 7.

## Technical Validation

### Analytic approach

#### Interrater reliabilities of items

We estimated interrater reliabilities of all 24 items. Human personality researchers have traditionally not examined the interrater reliabilities of individual items, partly because so much of this research was based on self-report data, but also because the reliability of individual items will tend to be quite modest, whereas those of dimensions, because they are based on multiple traits, are higher^56^. However, this extra caution is probably warranted in studies of nonhuman animals given that it is a relatively new field.

For the Kasekela and Mitumba communities we obtained two intraclass correlation coefficients (*ICC*s)^60^. The first, *ICC*(3,1), indicates the expected reliability of ratings from a single rater. The second, *ICC*(3,*k*), indicates the expected reliability of the mean ratings across *k* raters. Intraclass correlation coefficients are computed using mean squares obtained by fitting a linear model that predicts trait score by subject ID, the rater ID, and the subject×rater interaction, which is the error term.

*ICC*(3,1) is defined as:

$$\frac{MS_{B}-MS_{E}}{MS_{B}+\left(k-1\right)MS_{E}}$$

where *MS*_*B*_ refers to the mean square estimate for between-subjects term, *MS*_*E*_ refers to the mean square for the error term, and *k* refers to the mean number of raters per chimpanzee.

*ICC*(3,*k*) is defined as:

$$\frac{MS_{B}-MS_{E}}{MS_{B}}$$

where *MS*_*B*_ and *MS*_*E*_ are interpreted as before. Because *ICC*(3,*k*) estimates represent the reliability of ratings aggregated across multiple observations, they will be higher than the *ICC*(3,1) estimates, which, by definition, include variation in how each rater rates each chimpanzee, i.e., the error term, weighted by the number of raters minus one, in the denominator.

Most intraclass correlation coefficients range from 0 to 1. However, in cases where the error variance is greater than the subject variance, they can be negative. For our study, we only retained items that had reliabilities greater than 0 in both communities. This is a liberal criterion, but it is appropriate for item-level data because, as noted before, the amount of error variance associated with a single item is higher than the amount of error variance associated with component or factor scores that represent personality dimensions, as the latter are comprised of multiple traits^56^. A similar approach has been applied in previous studies of primates, such Weiss *et al.*^41^

#### Data reduction

To understand the personality dimensions that the correlations between the items define, we used exploratory factor analysis and principal components analysis. Both methods reduce large numbers of variables into a smaller number of dimensions. These methods are related procedures but differ in that *factors* are latent variables, i.e., variables that are not directly observed, and are modeled from the variables entered into the analysis, whereas *components* are mathematical summaries of the correlations among the variables entered into the analysis^61^. We used both methods, because the degree to which they yield similar dimensions indicates the degree to which methodological artifacts, such as unmeasured sources of error, influence the structure. In both cases, we analyzed only items with an interrater reliability greater than 0 in both communities.

The first step of either exploratory factor analysis or principal components analysis is to identify the number of dimensions that define the correlations among the variables. The second step of these analyses is to extract these dimensions, that is, to identify the size and direction of the associations between the individual variables and each of the dimensions. The third step of these analyses is to determine whether the dimensions are correlated with one another or whether they are mostly independent of one another. The fourth step is to interpret these dimensions, that is, to try to understand what underlying personality dimensions, if any, they describe. For example, if a dimension describes a grouping of traits all or most of which are related to interpersonal interactions, one might consider that trait to be a measure of extraversion or sociability. This is an important step, because it is possible that one or more dimensions cannot be interpreted in terms of a clear underlying behavioral tendency, suggesting that these dimensions reflect capitalization on chance or correlated error variance.

As previous studies of chimpanzees identified six dimensions^41,44^, we first examined whether extracting six dimensions *a priori* yielded interpretable personality dimensions. In addition, we used two exploratory methods that are commonly used together to determine the number of dimensions. The first involved creating a scree plot in which the eigenvalues (the amount of variance explained by each component or factor) are plotted against the component or factor number. We then inspected the scree plot to determine how many dimensions account for a substantial portion of variance^62^. The second involved using parallel analysis. In the present study, we conducted our parallel analysis by obtaining eigenvalues by sampling with replacement from the actual data and from randomly generated data that had the same number of subjects and number of variables as the actual data. After 1,000 iterations, we determined whether the amount of variance that each dimension explains in the actual data is greater than what one would expect by chance at the 95th percentile derived via parallel analysis^63^.

After forcing the extraction of six dimensions or extracting the number of dimensions based on our inspection of the scree plot and the results of the parallel analysis, we determined the degree to which pairs of dimensions were correlated. This guided our choice as to whether to use correlated dimensions or orthogonal dimensions in further analyses. To do this we first used an oblique (promax) rotation that allows the dimensions to be correlated with one another. If these correlations were relatively high, we used these dimensions in our further analyses. On the other hand, if these correlations were relatively low, we applied an orthogonal (varimax) rotation to these dimensions, which forces the correlations between dimensions to equal zero, and used these dimensions in our further analyses. Following studies of humans^64^ and chimpanzees^41^, to interpret the dimensions we defined factor or component loadings (the association between a dimension and the items) as salient (or meaningful) if they were equal to or greater than |0.4|. If, for any of these analyses, a large proportion of dimensions could not be interpreted in a straightforward manner based on previous studies of chimpanzee personality or behavior, we ignored those results and tried another means of dimension reduction or rotation. Finally, after determining the most suitable personality structure for this sample, as in other studies of wild nonhuman primates^4,10,18^, we used unit weighting to generate scores on each dimension^61^. This involves assigning a weight of +1 to items with salient loadings that were positive, a −1 to items with salient loadings that were negative, and a 0 to all other loadings. If an item had two or more salient loadings, it was assigned to the dimension onto which it had the highest absolute loading.

#### Interrater reliabilities of personality dimensions

We estimated the interrater reliabilities of the personality dimensions using *ICC*(3,1) and *ICC*(3,*k*).

#### Correlations between ratings from 1973 and ratings from the present study

We used Pearson correlations to compare the present personality ratings data to personality ratings data on the EPI that were collected in July and August 1973 (ref. 7). This enabled us to simultaneously test the degree to which wild chimpanzee personality was stable over time and the degree to which HPQ dimensions exhibited construct validity.

Ratings of 24 chimpanzees were made on the EPI by seven researchers (including undergraduates, graduate students and post-doctoral researchers) who were conducting research on the chimpanzees at Gombe National Park and who had known the chimpanzees for anywhere from several months to several years^7^. The EPI presents pairs of emotions and asks raters to indicate which of the two emotions better characterizes a chimpanzee. The ratings on the EPI items were then used to generate eight continuous variables representing the Aggressive, Distrustful, Controlled, Gregarious, Depressed, Timid, Trustful, and Dyscontrolled dimension for each chimpanzee. The EPI data for 23 of these chimpanzees are shown in Tables 2 and 3 of the 1978 paper^7^. The data for the 24th chimpanzee, Passion was not included in the 1978 paper, presumably because her personality ratings were so different from the other chimpanzees, but data on her personality are presented on page 208 and in Fig. 1 of the 1991 paper^8^.

The 24 chimpanzees that had been rated on the EPI were also rated on the HPQ (Supplementary Table 1). To test the degree to which HPQ dimensions exhibited construct validity, that is whether the EPI and HPQ dimensions that assessed similar constructs were correlated and whether EPI and HPQ dimensions that assessed different constructs were not correlated^35^, we examined the correlation coefficients between each of the eight EPI dimension scores and unit-weighted HPQ dimension scores. Given the similarities between human and chimpanzee personality dimensions^44^, we interpreted these correlations in light of what one would expect based on associations found in previous studies. We focused on the best available evidence on associations between (a) trait adjectives and the human Five-Factor Model^45,65^ and (b) lower-order personality dimensions (facets) and their parent dimensions on a gold standard measure of the human Five-Factor Model^66^.

For these analyses, in addition to reporting correlation coefficients and *P*-values, we noted which correlations were ≥|0.4| as this represents a medium to large effect size^67^, and is the mean of 40 year retest correlations reported in a meta-analysis of human personality studies^68^. Because personality in most of the human studies reported in this meta-analysis are based on self-reports using the same or very similar instruments, this criterion is conservative.

### Results

#### Item interrater reliabilities

The interrater reliabilities for chimpanzees in the Kasekela community and Mitumba community are presented in Table 1. The reliabilities of the ratings of the Kasekela chimpanzees were based on 318 ratings of 106 chimpanzees who were each rated by at least 2 of the 12 Tanzanian field assistants. These chimpanzees included two females (Q450 and Y235) who were observed in both communities and who were rated by more than one field assistant in Kasekela, but not a third female (E131) who had also been observed in both communities, because this third female was rated by only one field assistant in Kasekela. The interrater reliabilities for chimpanzees in the Mitumba community were based on 141 ratings of 24 chimpanzees by 6 field assistants. This sample included the females E131, Q450, and Y235, who were observed in both communities and rated by more than one field assistant in Mitumba.

As expected, the reliabilities of the items were modest, but they were within the range, albeit the low end, of what has been found in previous studies of chimpanzees. Although the overall reliabilities were similar between ratings from the Kasekela and Mitumba chimpanzees, the specific items that had relatively high and relatively low reliabilities varied some. Items with higher reliabilities from ratings of Kasekela chimpanzees were related to social interactions, including, probably, competition for status, but also sociopositive displays, such as sympathy for other chimpanzees. These items also included those related to exploratory behavior. Items with lower reliabilities included those related to emotional stability and vigilance, but also items describing the extent to which chimpanzees were predictable, reliable, and directed in their behavior. Ratings of Mitumba chimpanzees that had high reliabilities were items that described social interactions, both related to competition for status, but also the degree to which individuals interacted with conspecifics and their degree of exploratory behavior. Items from this group that had relatively low reliabilities, included those related to sociopositive behavior, but also those related to emotional stability and vigilance, and those describing the extent to which chimpanzees were predictable, reliable, and directed.

Based on these results, we excluded the items ‘thoughtless’, ‘unemotional’, ‘vulnerable’, ‘conventional’, and ‘cool’ from further analyses because these items had reliabilities equal to or below zero in the Kasekela sample, the Mitumba sample, or in both samples. We recommend that others using these data do the same.

#### Factor analyses

Our forced six factor solution with a promax rotation revealed correlations between pairs of factors ranging from |0.04| to |0.64| (mean |*r*|=0.24), with two being large (*r*≥|0.5|) and two being medium (|0.3|≤*r*<|0.5|) in size. These correlations are larger than those found in previous chimpanzee personality studies, e.g., mean |*r*|=0.13 (ref. 44). A varimax rotation that constrained correlations between factors to equal zero resulted in more than one loading on each factor. We determined the degree to which the promax and varimax solutions were congruent and found that the congruences of three factors were below 0.95, suggesting that there were some differences between the two solutions. These two findings therefore suggest that we should interpret the promax rotated factors because the factors were substantially correlated and yielded some differences in their meaning. In Table 2 we show the item loadings that make up the six promax-rotated factors and identify how the items in these factors relate to the dimensions identified in previous studies. On the basis of the item loadings for factors I, II, III, and VI we suggest that these factors could reasonably be interpreted as the Dominance, Extraversion, Openness, and Agreeableness dimensions, respectively, that have been identified in previous studies^41,44,47^. The item loadings for factor IV suggest that this factor described a tendency for chimpanzees who appear to be active to also appear to be predictable and individualistic. The items loading onto factor V describe tendencies to appear sensitive to other chimpanzees’ feelings, needs, and emotions, and to appear decisive in their own actions and behavior. The varimax-rotated structure is presented in Supplementary Table 3.

Our inspection of the scree plot and the results of a parallel analysis indicated that there were four factors. A promax rotation of these factors yielded an ultra-Heywood case, that is an absolute factor loading greater than or equal to 1, and indicates that this rotated structure was unacceptable and should be discarded. When these factors were subjected to a varimax rotation, the factors were not easily interpretable: factor I reflected aspects of high Dominance, Agreeableness, Neuroticism, and Openness; factor II reflected aspects of low Dominance, high Extraversion, high Agreeableness, and high Openness; factor III reflected aspects of high and low Extraversion and high and low Conscientiousness; and factor IV reflected low Neuroticism and high Agreeableness. We therefore disregarded these results, though we present this structure in Supplementary Table 4.

#### Principal components analyses

A forced six-component solution with promax-rotated components showed correlations between components similar to those typically seen in studies of chimpanzees^44^. These correlations ranged from |0.01| to |0.40| (mean |*r*|=0.14), with 3 of the 15 being medium in size. Of the components, the fifth was defined by a single item, indicating that the six-component solution with a promax rotation should be disregarded. These results are presented in Supplementary Table 5. Of the varimax-rotated components, the sixth was defined by a single item, indicating that the six-component solution with a varimax rotation should be disregarded. These results are presented in Supplementary Table 6.

The parallel analysis and scree plot indicated that there were four components. We therefore extracted four components and rotated them using the promax and varimax procedures. Both rotations yielded groupings resembling those derived from the varimax rotation of four factors (Supplementary Table 4), and so, like those factors, these components were not easily interpretable and disregarded. These promax- and varimax-rotated structures are presented in Supplementary Tables 7 and 8, respectively.

#### Summary of data reduction results

The dimensions described in our data reduction analyses differed depending on the approach used for data reduction (exploratory factor analysis versus principal components analysis) and factor rotation (varimax versus promax), and one solution, the promax rotation of four factors, yielded a Heywood case. The two analyses in which six factors were extracted did not reveal dimensions defined by single items, and so there were no problems with these analyses (Table 2 and Supplementary Table 3). These factor analyses also yielded four dimensions that somewhat resembled those found in other studies^41,44,47^.

Studies of chimpanzees^41,44,47^ and other nonhuman primates^10,48,69^ found that the choice of method for data reduction and the decision of whether to allow dimensions to correlate, has little to no effect on what items make up the dimensions. That was not the case in our present analyses. The difference between the present results and those of previous studies in this regard are not likely attributable to differences between the populations, but are more likely to be the result of our use of a brief questionnaire. This is because, for any given sample size, smaller item-to-factor ratios yield less stable factor structures^70^. Given that we did not find a personality structure that was consistent across analytic methods, for all further analyses, we computed unit-weighted scores based on the definitions of the six chimpanzee personality dimensions identified in a previous study that used the full length HPQ^41^. We provide these scores in the publicly available dataset.

#### Interrater reliabilities of dimensions

The interrater reliability estimates of the six personality dimensions were based on the same number of ratings, chimpanzees, and raters as in the analyses of the interrater reliabilities of the items (Table 3). The median of the *ICC*(3,1) (When comparing interrater reliabilities across different samples or populations that have different numbers of raters per individual, one should use *ICC*(3,1) estimates because they are not sensitive to differences in study design.) estimates for the Kasekela chimpanzees (0.18) was only slightly lower than that for the Mitumba chimpanzees (0.22).

Despite this overall similarity, however, there were differences at the level of dimensions. The *ICC*(3,1) estimates of Dominance, Neuroticism, and Openness were higher in Mitumba and the *ICC*(3,1) estimates of Extraversion, Conscientiousness and Agreeableness were higher in Kasekela. Compared to previous studies, the interrater reliabilities of the six dimensions in the Kasekela community were clearly lower for Dominance, Extraversion, and Conscientiousness. The interrater reliabilities of Agreeableness, Neuroticism, and Openness were more comparable to the reliabilities identified in previous studies. Compared to the interrater reliabilities derived in previous studies, those of the six factors in the Mitumba community were lower for Extraversion, Conscientiousness, and Agreeableness, but comparable for Dominance, Neuroticism, and Openness.

#### Correlations between EPI and HPQ dimensions

Of the 48 correlations between the eight EPI dimensions derived from ratings in 1973 and the six HPQ dimensions derived from ratings by Tanzanian field assistants in 2010, nine were statistically significant and greater than |0.4| (Table 4). The HPQ dimensions Dominance, Extraversion, Agreeableness, Neuroticism, and Openness had correlations that were significantly associated with at least one EPI dimension. The HPQ dimension of Conscientiousness was not significantly associated with any of the EPI dimensions and did not have a correlation exceeding |0.4| with any EPI dimension.

Of the significant correlations, six were consistent with previous studies. The positive correlation between EPI Trustful and HPQ Agreeableness is consistent with the fact that trust is associated with high Agreeableness^45,65^ and is a facet of Agreeableness^66^. The negative correlations between EPI Timid and both HPQ Dominance and HPQ Extraversion are consistent with the fact that the traits like timid and submissive define low Extraversion in humans^45,65^, and that assertiveness is a facet of human Extraversion^66^. The positive association between EPI Aggressive and HPQ Neuroticism is consistent with the fact that, in humans, the experience of anger and hostile emotions is a facet of Neuroticism^66^. The positive association between EPI Gregarious and HPQ Extraversion is consistent with the fact that gregariousness defined high Extraversion in one study^45^, and traits associated with gregariousness, i.e., affectionate, friendly, talkative, joiner, warm, not lonely, and person-oriented, defined high Extraversion in another study^65^. Moreover, gregariousness is a facet of human Extraversion^66^. The negative association between EPI Depressed and HPQ Agreeableness is consistent with the finding in some studies that human Agreeableness is associated with being genial and cheerful^45^.

Two correlations differed from expectations for humans but fit with expectations for chimpanzees. The first was the negative association between EPI Timid and HPQ Openness and the second was the positive association between EPI Gregarious and HPQ Openness. In humans, Openness is not typically associated with gregariousness or timidity. However, these correlations are consistent with the fact that two items that define HPQ Openness partly refer to interactions with conspecifics. For example, the HPQ defines the item ‘curious’ as ‘Subject has a desire to see or know about objects, devices, or *other chimpanzees*. This includes a desire to know about the affairs of *other chimpanzees* that do not directly concern the subject.’ (emphases added). Thus, because these chimpanzees are more willing to expose themselves to risks from the environment or other chimpanzees, these correlations make sense in this case.

The single correlation that was unexpected based on previous studies of both humans and chimpanzees was the positive correlation between EPI Gregarious and HPQ Agreeableness. In humans, gregariousness is strongly associated with Extraversion. However, given that Agreeableness and gregariousness are associated with interactions with others, this correlation again makes sense for chimpanzees. As Agreeableness is related to the quality of social interactions, for example, whether they are characterized by friendliness, helpfulness, and getting along versus being competitive and selfish, the association between EPI Gregarious and HPQ Agreeableness suggests that EPI Gregarious measures these characteristics as well as the degree to which individuals interact with other chimpanzees.

Finally, some associations that one would have expected based on human personality studies were not statistically significant or did not meet our criteria. For one, given the association between the trust and Agreeableness across studies of human personality^45,65,66^, we would have expected to find a negative association between EPI Distrustful and HPQ Agreeableness. However, although this correlation was negative, it was far below |0.4| and it was also not statistically significant. Likewise, given their definitions, we would have expected EPI Controlled and Dyscontrolled to be associated with HPQ Conscientiousness or HPQ Neuroticism. In humans, self-discipline (similar to EPI Controlled), is associated with Conscientiousness^45,65,66^, while impulsivity (similar to EPI Dyscontrolled) is associated with Neuroticism^65,66^. For EPI Controlled and HPQ Conscientiousness and Neuroticism, the correlations were in the expected directions but did not meet our criteria. In both cases, however, EPI Dyscontrolled had negligible, non-significant correlations in the opposite of the expected direction with HPQ Conscientiousness and with HPQ Neuroticism. In addition, the fact that the correlation between the EPI Aggressive dimension and HPQ Dominance dimension did not meet either criteria is surprising given that EPI Aggressive was associated with higher dominance rank in Buirski *et al.*’s earlier study^7^. However, the correlation between these two variables is in the expected direction (positive) and the correlation is just a little below |0.4|. Overall, then, the convergent validity of the dimensions could be described as good and the discriminant validity could be described as excellent. These scales are thus largely measuring the constructs that they would be expected to measure based on their content.

## Usage Notes

We collected personality ratings of wild chimpanzees using a 24-item questionnaire that had been translated into Swahili. Of the 24 items, 19 met our threshold for interrater reliability. However, these ratings did not yield a personality structure that was consistent across different methods of identifying personality dimensions. Specifically, factor analysis and principal components analyses, and rotations that allowed dimensions to correlate (promax) or that fixed the correlations between dimensions to zero (varimax) yielded different sets of personality dimensions. Nonetheless, the factor analysis with a promax rotation in which six factors were extracted yielded four dimensions that somewhat resembled those described in previous studies of chimpanzees^41,44,47,55,71,72^. Furthermore, in 24 chimpanzees rated in 1973 and in 2010 we found good evidence for the retest reliability and construct validity of our measure. Out of 48 correlations, nine were medium to large, statistically significant (*P*<0.05), and consistent with the definitions of the dimensions in humans or in chimpanzees. There were cases where we expected to see medium to large correlations, but did not, e.g., an association between EPI Aggressive and HPQ Dominance (though in this case the correlation was in the expected direction and came close to meeting our criteria for inclusion). Considering that the EPI ratings were made nearly 40 years previously by expatriate undergraduate and graduate students and post-doctoral researchers and the more recent HPQ ratings were made by Tanzanian field assistants, this finding is remarkable.

As we noted in the Technical Validation section, some caution is warranted in the use of these data and in how results of future studies are to be interpreted. For one, although the interrater reliabilities of 19 of the 24 items and of the six dimensions met our threshold for inclusion in further analyses, they tended to be lower than those found in studies of chimpanzees in zoos, other captive settings, and in a naturalistic sanctuary^41,44,47,55^. However, these interrater reliabilities were within the range of interrater reliabilities found in studies of human personality^73,74^, and so we consider them acceptable.

The low interrater reliabilities in this study compared to previous studies of chimpanzees may be attributable to the fact that, counting ratings that were excluded, each field assistant rated many more chimpanzees (range: 21–43, mean=27.4) than did raters in previous studies: means=8.7 and 15.6 for chimpanzees housed in zoos and Yerkes National Primate Research Center, respectively^47^ and a mean of 10.15 for chimpanzees living in zoos, research centers, and a sanctuary in Japan^41^. This explanation is consistent with the finding that the interrater reliabilities of the mean ratings were higher for the Mitumba chimpanzees as, counting ratings that were excluded, field assistants who rated the chimpanzees belonging to that community had fewer chimpanzees to rate (median=23.5) than field assistants who rated the chimpanzees belonging to the Kasekela community (median=30.0). However, the increased rater response burden may not fully explain the lower interrater reliabilities as, in the study of chimpanzees housed in Yerkes National Primate Research Center, 2 of the 16 raters were responsible for rating 75 chimpanzees, and thus faced a very high response burden^47^, yet the interrater agreement found in this study was higher. Another factor that may explain the higher interrater reliabilities for Mitumba is that this community has been studied for a shorter time by a smaller group of field assistants, who have all known the Mitumba chimpanzees since at least 2003. Thus, both the set of chimpanzees, and the set of raters, are less heterogeneous for Mitumba than for Kasekela.

There are two other possible sources of greater heterogeneity in the ratings of the Kasekela chimpanzees than of the Mitumba chimpanzees. The first is that the Kasekela chimpanzees were rated in equal measure by both, field assistants who were relatively young and currently employed and by field assistants who were older and had been retired for many years. The second is that this sample was made up of about the same number of chimpanzees who had died before 2010, and thus were likely to have been rated by older, retired field assistants, and chimpanzees who were still living, which were more likely to be rated by the younger, currently employed field assistants. We used our data to conduct exploratory analyses to test both possibilities. We first compared the interrater reliabilities of single ratings between a subsample of four older, retired field assistants who rated a total of 34 chimpanzees and five younger, still employed field assistants who rated a total of 47 chimpanzees. Second, we compared a subsample of 57 deceased chimpanzees who had been rated by 12 field assistants with a subsample of 49 chimpanzees who were still alive, and who had been rated by 11 field assistants. We included only those chimpanzees that had been rated by two or more field assistants in these comparisons.

The results of both analyses are presented in Supplementary Table 9. Overall, older, retired field assistants provided more reliable ratings (median interrater reliability=0.25) than younger, currently employed field assistants (median interrater reliability=0.16). At the level of individual dimensions, the reliabilities for Conscientiousness, Neuroticism, and Openness were higher in the older group, the reliability of Extraversion was higher in the younger group, and the reliabilities of Dominance and Agreeableness were nearly identical across groups. Whether the chimpanzees rated were deceased or alive had no overall bearing on the reliabilities of the ratings (median interrater reliability=0.20 for both). At the domain level, however, the reliabilities for Conscientiousness and possibly Openness were higher in the deceased chimpanzees, the reliability of Agreeableness was higher in living chimpanzees, and the reliabilities of Dominance, Extraversion, and Neuroticism were nearly identical across groups. These results suggest that, perhaps because they worked with chimpanzees for considerably longer, the older, retired field assistants provided more reliable ratings than the younger, employed field assistants. Moreover, this advantage seemed most pronounced for dimensions describing characteristics that are possibly more subtle and rare, such as behaviors related to Conscientiousness. These findings support the view that the greater heterogeneity of the raters in the Kasekela group was responsible for the lower reliabilities in this group.

Another question concerning these data is why, unlike other studies^41,44,47,55^, our set of dimensions varied across different data reduction methods and means of rotating the structure. Several characteristics of the present study are likely responsible for this finding. These include (a) the heterogeneity of our raters (18 different men with greatly varying work experience), (b) the long time frame over which raters based their assessments of chimpanzees (up to 35 years, so longer than the lifespan of many chimpanzees), (c) the large number of chimpanzees rated by each rater (again, counting ratings that were excluded, range: 21–43; median=25.5 chimpanzees per rater), (d) the fact that ~49% of the chimpanzees were dead before we began to collect ratings with approximately ~71% of these deaths occurring at least 10 years before the ratings took place), **e)** that 8 of 18 of the field assistants had not been working with the chimpanzees in that community for one to six years because they retired, were no longer employed, or had switched communities, and **f)** that field assistants varied considerably in their total years of experience and in their years of overlap with particular chimpanzees. In addition to possibilities a through f, a major determinant of the stability of factor structure is the ratio of the number of items to the number of factors^70^. In the present study, this ratio was low, which could explain why we did not find a structure similar to that found repeatedly in other studies of chimpanzee personality^41,44,47,55^ or a more stable structure as has been found in studies of wild^4,18^ and semi-wild nonhuman primates^10^. Given these limitations, we cannot rule out the possibility that the personality structure in wild chimpanzees is the same as the personality structure found in captive populations.

Finally, although the small number of items makes it impossible to test this possibility, studies of humans suggest that cultural differences between raters may play a role in why we found differences between the personality dimensions in these chimpanzees and those in previous studies. For instance, a large study of personality found lower internal consistency reliabilities (a measure of how intercorrelated items within dimensions are) in African countries than in nine other world regions, and that one of the five dimensions (Openness) was not clearly represented^75^. A second large cross-cultural study found that anywhere between one to four of the five human dimensions were not clearly represented in four African countries^76^. A study of the Tsimane, a traditional forager-farmers community living in the Bolivian rainforest was also not able to replicate the five human dimensions found in so many other cultures^77^.

Although the present data require some caveats regarding reliability and personality structure, personality data on humans with similar limitations have been important for understanding personality evolution. For example, despite the inability to replicate the personality dimensions found in other populations, possibly because of translation issues or the need to devise another way for participants to respond to questions, a study using the data collected on the Tsimane revealed associations between personality dimensions and both fertility and health^78^. Similarly, despite using a 27-item measure of the five human dimensions that had low internal consistency reliabilities, one group of researchers. found associations between personality and fertility in traditional villages located in rural Senegal^79^.

Thus, despite these caveats, we consider the dataset we present here a valuable resource for personality research. The correlations between ratings from this study and personality measures assessed nearly 40 years earlier indicate the existence of stable personality traits in our study population of chimpanzees. The personality measures we derive here can therefore be used to examine associations with components of fitness and other variables in a long-lived mammal that has close phylogenetic ties to our own species, and are therefore a promising resource for researchers who seek to gain insights into the evolutionary bases of personality in chimpanzees, other nonhuman primates, and humans. We see four ways that these data can be used either by themselves or in combination with other data. First, follow-up studies can examine the long-term behavioral and fitness consequences of the personalities of chimpanzees who are still alive. Second, research may examine associations between personality, behavior and life-history variables that were collected before these ratings were obtained. To facilitate causal inferences, the findings of these studies could be followed by prospective studies. Third, using historical data on the population, the social environment, and other events at the time of birth or during ‘sensitive periods’ in development, researchers could determine the degree to which these events influence personality development in specific birth cohorts. Finally, these data will provide useful comparative data for studies of personality and fitness in modern humans^78–80^, and in chimpanzees, too.

Given the difficulties of identifying a stable personality structure in these data, one decision comparative studies will grapple with is whether to use the replicable personality structure obtained in previous studies^41,44,47^, one of the personality structures identified using the analyses presented here, or to define personality dimensions *a priori* to address their specific research question. Ultimately, this decision will rest on the research question(s). For example, although we prefer to use personality dimensions found in existing studies of chimpanzees, a behavioral ecologist may decide, based on the correlations between the HPQ and the EPI, to construct a measure of ‘boldness,’ by summing Dominance, Extraversion, Openness, and the inverse of Conscientiousness. Another approach may be to maximize the similarity of personality dimensions between this and other samples by using only items or groups of items that are similar across samples. For example, taking the six dimensions found earlier (Table 2) and the dimensions found in chimpanzees in Japan^41^, in both samples Dominance would be defined by the item dominant, Extraversion would be defined by the items sociable and solitary, (low) Conscientiousness would be defined by the items reckless and impulsive, Agreeableness would be defined by the items sympathetic and helpful, and Neuroticism would be defined by the item stable. Although gathering data on this population using the full-length questionnaire is a desirable goal for the future, studies of human personality have found major, replicable findings, such as association between Conscientiousness and longevity, using archival measures consisting of one or two items to represent each dimension^81^. Regardless of what researchers who use this data set decide to do, we strongly urge that those researchers preregister their studies to avoid ‘fishing expeditions’ in which they try multiple ways of combining traits until they find a significant result.

These data provide measures of the personalities of chimpanzees from a population that is well known for having distinctive personalities, but for which few quantitative data on personality were previously available. Considering the extensive long-term data on Gombe chimpanzees, we expect that making these data available will promote advances in the understanding of personality evolution in primates and other species, including humans.

## Additional information

**How to cite this article:** Weiss, A. *et al.* Personality in the chimpanzees of Gombe National Park. *Sci. Data* 4:170146 doi: 10.1038/sdata.2017.146 (2017).

**Publisher’s note:** Springer Nature remains neutral with regard to jurisdictional claims in published maps and institutional affiliations.

## Supplementary Material



Supplementary Information

## Figures and Tables

**Table 1:** Interrater reliabilities of ratings of the chimpanzees in the Kasekela and Mitumba communities.

**Item**	**Kasekela**		**Mitumba**	
	***ICC*****(3,1)**	***ICC*****(3,*****k***)	***ICC*****(3,1)**	***ICC*****(3,*****k***)
Dominant	0.40	0.66	0.42	0.81
Reckless	0.25	0.49	0.09	0.38
Excitable	0.24	0.49	0.39	0.79
Sociable	0.21	0.45	0.06	0.28
Sensitive	0.20	0.42	0.08	0.35
Sympathetic	0.19	0.42	0.17	0.55
Innovative	0.18	0.39	0.25	0.66
Active	0.17	0.38	0.16	0.53
Helpful	0.17	0.38	0.14	0.50
Inquisitive	0.17	0.38	0.23	0.63
Impulsive	0.15	0.35	0.21	0.60
Solitary	0.13	0.32	0.25	0.66
Inventive	0.13	0.31	0.12	0.44
Curious	0.12	0.30	0.15	0.50
Decisive	0.12	0.29	0.23	0.64
Stable	0.09	0.23	0.07	0.30
Cool	0.06	0.17	−0.05	−0.37
Individualistic	0.05	0.13	0.01	0.04
Predictable	0.04	0.12	0.05	0.24
Dependent/follower	0.04	0.11	0.16	0.54
Thoughtless	0.01	0.02	−0.03	−0.21
Vulnerable	0.00	−0.01	−0.04	−0.30
Conventional	−0.02	−0.05	−0.02	−0.12
Unemotional	−0.06	−0.22	0.00	−0.01
For the chimpanzees in the Kasekela community the mean number of raters per chimpanzee (*k*) was 3.00 (range: 2–5 raters per chimpanzee). For the chimpanzees in the Mitumba community the mean number of raters per chimpanzee was 5.88 (range: 3–7 raters per chimpanzee). For the Mitumba chimpanzee rated by the same rater twice we treated each rating as coming from the same rater.				

**Table 2:** Promax rotation of six factor solution

	**Factor**							
**Item**	**I**	**II**	**III**	**IV**	**V**	**VI**	***h***^**2**^	**Item’s loading in previous studies** *
Dominant	**0.87**	0.04	−0.19	0.01	0.24	−0.07	0.74	Dominance (+)
Reckless	**0.70**	0.09	−0.09	0.24	−0.04	−0.16	0.59	Conscientiousness (−)
Impulsive	**0.48**	−0.12	0.20	0.32	−0.01	−0.14	0.56	Conscientiousness (−)
Excitable	**0.47**	0.00	0.32	0.01	0.14	−0.10	0.63	Neuroticism (+)
Inventive	**0.46**	−0.10	0.13	−0.07	0.26	0.08	0.44	Openness (+)
Innovative	0.39	0.38	0.23	−0.22	−0.23	0.08	0.58	Openness (+)
Sociable	−0.08	**0.72**	0.26	−0.02	−0.01	−0.06	0.67	Extraversion (+)
Dependent/follower	0.00	**0.66**	−0.15	0.09	−0.14	0.09	0.42	Dominance (−)
Solitary	−0.01	−**0.45**	−0.16	0.27	−0.20	0.14	0.43	Extraversion (−)
Inquisitive	0.04	0.08	**0.87**	−0.04	−0.12	0.01	0.75	Openness (+)
Curious	−0.03	0.08	**0.70**	0.28	−0.08	0.05	0.56	Openness (+)
Active	−0.08	0.17	−0.01	**0.60**	**0.44**	−0.24	0.66	Extraversion (+)
Predictable	0.05	0.00	0.06	**0.59**	0.03	0.07	0.41	Conscientiousness (+)
Individualistic	0.15	−0.12	0.03	**0.55**	−0.16	0.03	0.35	Extraversion (−)
Decisive	0.15	0.03	−0.17	0.01	**0.67**	0.04	0.42	Dominance (+)
Sensitive	0.19	−0.37	0.16	−0.13	**0.65**	0.19	0.73	Agreeableness (+)
Sympathetic	−0.17	−0.07	0.01	−0.08	0.02	**0.60**	0.39	Agreeableness (+)
Helpful	0.32	0.39	−0.07	0.08	0.11	**0.56**	0.76	Agreeableness (+)
Stable	−0.13	0.09	0.08	0.21	0.21	0.34	0.28	Neuroticism (−)
Proportion of variance	0.14	0.09	0.09	0.08	0.08	0.05		
	Factor Correlations							
	I	II	III	IV	V			
	0.29					II		
	0.64	0.40				III		
	0.26	−0.04	0.09			IV		
	0.33	0.13	0.57	0.25		V		
	0.04	0.17	0.12	0.04	0.16	VI		
*h*^2^=Proportion of variance in each item accounted for by all six factors. Salient loadings (≥|0.4|) in boldface								

*Refers to the direction of the association between each item and chimpanzee personality dimensions identified in previous studies.

**Table 3:** Interrater reliabilities of personality dimensions in the Kasekela community, the Mitumba community, and interrater reliabilities of individual ratings obtained in previous studies.

**Dimension**	**Kasekela**		**Mitumba**		***ICC*****(3,1) estimates from previous studies**			
	***ICC*****(3,1)**	***ICC *****(3,*****k***)	***ICC*****(3,1)**	***ICC*****(3,*****k***)	**King & Figueredo (1997)**^44^	**King** ***et al*****. (2005)**^55^	**Weiss** ***et al*****. (2007)**^47^	**Weiss** ***et al*****. (2009)**^41^
Dominance	0.25	0.51	0.47	0.84	0.64	0.51	0.48	0.63
Extraversion	0.18	0.39	0.13	0.47	0.56	0.29	0.38	0.56
Conscientiousness	0.18	0.40	0.04	0.19	0.39	0.37	0.38	0.32
Agreeableness	0.18	0.40	0.12	0.46	0.33	0.23	0.22	0.48
Neuroticism	0.15	0.35	0.32	0.73	0.36	0.17	—	0.38
Openness	0.27	0.53	0.32	0.73	0.52	0.24	—	0.51
For the chimpanzees in the Kasekela community the mean number of raters per chimpanzee (*k*) was 3.00 (range: 2 to 5 raters per chimpanzee). For the chimpanzees in the Mitumba community the mean number of raters per chimpanzee (*k*) was 5.88 (range: 3 to 7 raters per chimpanzee). For the Mitumba chimpanzee rated by the same rater twice we treated each rating as coming from the same rater in these analyses.								

**Table 4:** Correlations between the six unit-weighted scores based on the definitions in Weiss, *et al.*^41^ and the eight Emotions Profile Index dimensions from Buirski, *et al.*^7^

	**Hominoid Personality Questionnaire**											
	**Dominance**		**Extraversion**		**Conscientiousness**		**Agreeableness**		**Neuroticism**		**Openness**	
**Emotions Profile Index**	***r***	***P***	***r***	***P***	***r***	***P***	***r***	***P***	***r***	***P***	***r***	***P***
Trustful	−0.34	0.104	−0.16	0.442	0.32	0.122	**0.41**	**0.045**	−0.40*	0.055	−0.12	0.581
Distrustful	0.31	0.146	0.24	0.268	−0.19	0.378	−0.08	0.725	0.22	0.308	0.38	0.068
Timid	−**0.65**	**0.001**	−**0.59**	**0.002**	0.38	0.066	−0.02	0.940	−0.22	0.292	−**0.53**	**0.007**
Aggressive	0.38	0.067	−0.02	0.924	−0.08	0.694	−0.24	0.268	**0.41**	**0.045**	0.15	0.495
Controlled	−0.38	0.067	−0.11	0.622	0.12	0.571	−0.07	0.755	−0.04	0.870	−0.39	0.062
Dyscontrolled	−0.09	0.685	0.20	0.344	0.04	0.837	0.09	0.685	−0.31	0.134	0.07	0.741
Gregarious	0.29	0.163	**0.51**	**0.010**	−0.13	0.532	**0.50**	**0.013**	0.08	0.710	**0.51**	**0.011**
Depressed	0.03	0.876	−0.22	0.311	−0.15	0.479	−**0.43**	**0.037**	−0.02	0.923	−0.35	0.092
*N*=24. Statistically significant (*P*<0.05) Pearson correlation coefficients are in boldface.												

*Before rounding, *r*<0.4.
