# Supplementary material for: Personality in the chimpanzees of Gombe National Park
Source: Sci Data. 2017 Oct 24;4:170146. doi: 10.1038/sdata.2017.146 (PMC5654364; doi:10.1038/sdata.2017.146)
Supplement: Supplementary Information [file sdata2017146-s2.pdf]

Supplementary Materials

Document	Pages
File 1: Full-length English-language version of the Hominoid Personality Questionnaire	1-13
File 2: Full-length Swahili-language version of the Hominoid Personality Questionnaire	14-25
File 3: Brief Swahili-language version of the Hominoid Personality Questionnaire	26-31
Table S1: Raters and chimpanzees of the Kasekela community. Shaded chimpanzee codes indicate those rated on the HPQ and the Emotions Profile Index. Raters without an end date are still employed as field assistants	32
Table S2: Raters and chimpanzees of the Mitumba community. The rater without an end date is still employed as a field assistant	33
Table S3: Varimax rotation of six factor solution	34
Table S4: Varimax rotation of four factor solution	35
Table S5: Promax rotation of six component solution	36
Table S6: Varimax rotation of six component solution	37
Table S7: Promax rotation of four component solution	38
Table S8: Varimax rotation of four component solution	39
Table S9: Interrater reliabilities of chimpanzees in the Kasekela community separated by employment status of raters and whether the chimpanzees were alive or dead	40

CHIMPANZEE PERSONALITY TRAIT ASSESSMENT

Chimpanzee personality assessments can be made with this questionnaire by assigning a numerical score for all of the personality traits listed on the following pages. Make your judgments on the basis of your own understanding of the trait guided by the short clarifying definition following each trait. The chimpanzee's own behaviors and interactions with other chimpanzees should be the basis for your numerical ratings. Use your own subjective judgment of typical chimpanzee behavior to decide if the chimpanzee you are scoring is above, below, or average for a trait. The following seven point scale should be used to make your ratings.

1. **Displays either total absence or negligible amounts of the trait.**
2. **Displays small amounts of the trait on infrequent occasions.**
3. **Displays somewhat less than average amounts of the trait.**
4. **Displays about average amounts of the trait.**
5. **Displays somewhat greater than average amounts of the trait.**
6. **Displays considerable amounts of the trait on frequent occasions.**
7. **Displays extremely large amounts of the trait.**

Please give a rating for each trait even if your judgment seems to be based on a purely subjective impression of the chimpanzee and you are somewhat unsure about it. Indicate your rating by placing a cross in the box underneath the chosen number. [×]

Finally, do not discuss your rating of any particular chimpanzee with anyone else. As explained in the handout accompanying this questionnaire, this restriction is necessary in order to obtain valid reliability coefficients for the traits.

CHIMPANZEE PERSONALITY TRAIT ASSESSMENT

Chimpanzee's full name:________________________________

Rater's full name:________________________________

Date (Mon/Day/Yr):________________________________

FEARFUL: Subject reacts excessively to real or imagined threats by displaying behaviors such as screaming, grimacing, running away or other signs of anxiety or distress.

least	1	2	3	4	5	6	7	most
	☐	☐	☐	☐	☐	☐	☐	

DOMINANT: Subject is able to displace, threaten, or take food from other chimpanzees. Or subject may express high status by decisively intervening in social interactions.

least	1	2	3	4	5	6	7	most
	☐	☐	☐	☐	☐	☐	☐	

PERSISTENT: Subject tends to continue in a course of action, task, or strategy for a long time or continues despite opposition from other chimpanzees.

least	1	2	3	4	5	6	7	most
	☐	☐	☐	☐	☐	☐	☐	

CAUTIOUS: Subject often seems attentive to possible harm or danger from its actions. Subject avoids risky behaviors.

least	1	2	3	4	5	6	7	most
	☐	☐	☐	☐	☐	☐	☐	

STABLE: Subject reacts to its environment including the behavior of other chimpanzees in a calm, equable, way. Subject is not easily upset by the behaviors of other chimpanzees.

least most

1	2	3	4	5	6	7
☐	☐	☐	☐	☐	☐	☐

AUTISTIC: Subject often displays repeated, continuous, and stereotyped behaviors such as rocking or self clasping.

least most

1	2	3	4	5	6	7
☐	☐	☐	☐	☐	☐	☐

CURIOUS: Subject has a desire to see or know about objects, devices, or other chimpanzees. This includes a desire to know about the affairs of other chimpanzees that do not directly concern the subject.

least most

1	2	3	4	5	6	7
☐	☐	☐	☐	☐	☐	☐

THOUGHTLESS: Subject often behaves in a way that seems imprudent or forgetful.

least most

1	2	3	4	5	6	7
☐	☐	☐	☐	☐	☐	☐

STINGY/GREEDY: Subject is excessively desirous or covetous of food, favored locations, or other resources in the enclosure. Subject is unwilling to share these resources with others.

least							most
	1	2	3	4	5	6	7
	☐	☐	☐	☐	☐	☐	☐

JEALOUS: Subject is often troubled by others who are in a desirable or advantageous situation such as having food, a choice location, or access to social groups. Subject may attempt to disrupt activities of advantaged chimpanzees.

least							most
	1	2	3	4	5	6	7
	☐	☐	☐	☐	☐	☐	☐

INDIVIDUALISTIC: Subject's behavior stands out compared to that of the other individuals in the group. This does not mean that it does not fit or is incompatible with the group.

least							most
	1	2	3	4	5	6	7
	☐	☐	☐	☐	☐	☐	☐

RECKLESS: Subject is rash or unconcerned about the consequences of its behaviors.

least							most
	1	2	3	4	5	6	7
	☐	☐	☐	☐	☐	☐	☐

SOCIABLE: Subject seeks and enjoys the company of other chimpanzees and engages in amicable, affable, interactions with them.

least								most
	1	2	3	4	5	6	7	
	☐	☐	☐	☐	☐	☐	☐	

DISTRACTIBLE: Subject is easily distracted and has a short attention span.

least								most
	1	2	3	4	5	6	7	
	☐	☐	☐	☐	☐	☐	☐	

TIMID: Subject lacks self confidence, is easily alarmed and is hesitant to venture into new social or non-social situations.

least								most
	1	2	3	4	5	6	7	
	☐	☐	☐	☐	☐	☐	☐	

SYMPATHETIC: Subject seems to be considerate and kind towards others as if sharing their feelings or trying to provide reassurance.

least								most
	1	2	3	4	5	6	7	
	☐	☐	☐	☐	☐	☐	☐	

PLAYFUL: Subject is eager to engage in lively, vigorous, sportive, or acrobatic behaviors with or without other chimpanzees.

least								most
	1	2	3	4	5	6	7	
	☐	☐	☐	☐	☐	☐	☐	

SOLITARY: Subject prefers to spend considerable time alone not seeking or avoiding contact with other chimpanzees.

least								most
	1	2	3	4	5	6	7	
	☐	☐	☐	☐	☐	☐	☐	

VULNERABLE: Subject is prone to be physically or emotionally hurt as a result of dominance displays, highly assertive behavior, aggression, or attack by another chimpanzee.

least								most
	1	2	3	4	5	6	7	
	☐	☐	☐	☐	☐	☐	☐	

INNOVATIVE: Subject engages in new or different behaviors that may involve the use of objects or materials or ways of interacting with others.

least								most
	1	2	3	4	5	6	7	
	☐	☐	☐	☐	☐	☐	☐	

ACTIVE: Subject spends little time idle and seems motivated to spend considerable time either moving around or engaging in some overt, energetic behavior.

least								most
	1	2	3	4	5	6	7	
	☐	☐	☐	☐	☐	☐	☐	

HELPFUL: Subject is willing to assist, accommodate, or cooperate with other chimpanzees.

least								most
	1	2	3	4	5	6	7	
	☐	☐	☐	☐	☐	☐	☐	

BULLYING: Subject is overbearing and intimidating towards younger or lower ranking chimpanzees.

least	1	2	3	4	5	6	7	most
	☐	☐	☐	☐	☐	☐	☐	

AGGRESSIVE: Subject often initiates fights or other menacing and agonistic encounters with other chimpanzees.

least	1	2	3	4	5	6	7	most
	☐	☐	☐	☐	☐	☐	☐	

MANIPULATIVE: Subject is adept at forming social relationships for its own advantage, especially using alliances and friendships to increase its social standing. Chimpanzee seems able and willing to use others.

least	1	2	3	4	5	6	7	most
	☐	☐	☐	☐	☐	☐	☐	

GENTLE: Subject responds to others in an easy-going, kind, and considerate manner. Subject is not rough or threatening.

least	1	2	3	4	5	6	7	most
	☐	☐	☐	☐	☐	☐	☐	

AFFECTIONATE: Subject seems to have a warm attachment or closeness with other chimpanzees. This may entail frequently grooming, touching, embracing, or lying next to others.

least	1	2	3	4	5	6	7	most
	☐	☐	☐	☐	☐	☐	☐	

EXCITABLE: Subject is easily aroused to an emotional state. Subject becomes highly aroused by situations that would cause less arousal in most chimpanzees.

least								most
	1	2	3	4	5	6	7	
	☐	☐	☐	☐	☐	☐	☐	

IMPULSIVE: Subject often displays some spontaneous or sudden behavior that could not have been anticipated. There often seems to be some emotional reason behind the sudden behavior.

least								most
	1	2	3	4	5	6	7	
	☐	☐	☐	☐	☐	☐	☐	

INQUISITIVE: Subject seems drawn to new situations, objects, or animals. Subject behaves as if it wishes to learn more about other chimpanzees, objects, or persons within its view.

least								most
	1	2	3	4	5	6	7	
	☐	☐	☐	☐	☐	☐	☐	

SUBMISSIVE: Subject often gives in or yields to another chimpanzee. Subject acts as if it is subordinate or of lower rank than other chimpanzees.

least								most
	1	2	3	4	5	6	7	
	☐	☐	☐	☐	☐	☐	☐	

COOL: Subject seems unaffected by emotions and is usually undisturbed, assured, and calm.

least								most
	1	2	3	4	5	6	7	
	☐	☐	☐	☐	☐	☐	☐	

DEPENDENT/FOLLOWER: Subject often relies on other chimpanzees for leadership, reassurance, touching, embracing and other forms of social support.

least								most
	1	2	3	4	5	6	7	
	☐	☐	☐	☐	☐	☐	☐	

IRRITABLE: Subject often seems in a bad mood or is impatient and easily provoked to anger exasperation and consequent agonistic behavior.

least								most
	1	2	3	4	5	6	7	
	☐	☐	☐	☐	☐	☐	☐	

UNPERCEPTIVE: Subject is slow to respond or understand moods, dispositions, or behaviors of others.

least								most
	1	2	3	4	5	6	7	
	☐	☐	☐	☐	☐	☐	☐	

PREDICTABLE: Subject's behavior is consistent and steady over extended periods of time. Subject does little that is unexpected or deviates from its usual behavioral routine.

least								most
	1	2	3	4	5	6	7	
	☐	☐	☐	☐	☐	☐	☐	

DECISIVE: Subject is deliberate, determined, and purposeful in its activities.

least							most
	1	2	3	4	5	6	7
	☐	☐	☐	☐	☐	☐	☐

DEPRESSED: Subject does not seek out social interactions with others and often fails to respond to social interactions of other chimpanzees. Subject often appears isolated, withdrawn, sullen, brooding, and has reduced activity.

least							most
	1	2	3	4	5	6	7
	☐	☐	☐	☐	☐	☐	☐

CONVENTIONAL: Subject seems to lack spontaneity or originality. Subject behaves in a consistent manner from day to day and stays well within the social rules of the group.

least							most
	1	2	3	4	5	6	7
	☐	☐	☐	☐	☐	☐	☐

SENSITIVE: Subject is able to understand or read the mood, disposition, feelings, or intentions of other chimpanzees often on the basis of subtle, minimal cues.

least							most
	1	2	3	4	5	6	7
	☐	☐	☐	☐	☐	☐	☐

DEFIANT: Subject is assertive or contentious in a way inconsistent with the usual dominance order. Subject maintains these actions despite unfavorable consequences or threats from others.

least							most
	1	2	3	4	5	6	7
	☐	☐	☐	☐	☐	☐	☐

INTELLIGENT: Subject is quick and accurate in judging and comprehending both social and non-social situations. Subject is perceptive and discerning about social relationships.

least							most
	1	2	3	4	5	6	7
	☐	☐	☐	☐	☐	☐	☐

PROTECTIVE: Subject shows concern for other chimpanzees and often intervenes to prevent harm or annoyance from coming to them.

least							most
	1	2	3	4	5	6	7
	☐	☐	☐	☐	☐	☐	☐

QUITTING: Subject readily stops or gives up activities that have recently been started.

least							most
	1	2	3	4	5	6	7
	☐	☐	☐	☐	☐	☐	☐

INVENTIVE: Subject is more likely than others to do new things including novel social or non-social behaviors. Novel behavior may also include new ways of using devices or materials.

least								most
	1	2	3	4	5	6	7	
	☐	☐	☐	☐	☐	☐	☐	

CLUMSY: Subject is relatively awkward or uncoordinated during movements including but not limited to walking, acrobatics, and play.

least								most
	1	2	3	4	5	6	7	
	☐	☐	☐	☐	☐	☐	☐	

ERRATIC: Subject is inconsistent, indefinite, and widely varying in its behavior and moods.

least								most
	1	2	3	4	5	6	7	
	☐	☐	☐	☐	☐	☐	☐	

FRIENDLY: Subject often seeks out contact with other chimpanzees for amiable, genial activities. Subject infrequently initiates hostile behaviors towards other chimpanzees.

least								most
	1	2	3	4	5	6	7	
	☐	☐	☐	☐	☐	☐	☐	

ANXIOUS: Subject often seems distressed, troubled, or is in a state of uncertainty.

least								most
	1	2	3	4	5	6	7	
	☐	☐	☐	☐	☐	☐	☐	

LAZY: Subject is relatively inactive, indolent, or slow moving and avoids energetic activities.

least								most
	1	2	3	4	5	6	7	
	☐	☐	☐	☐	☐	☐	☐	

DISORGANIZED: Subject is scatterbrained, sloppy, or haphazard in its behavior as if not following a consistent goal.

least								most
	1	2	3	4	5	6	7	
	☐	☐	☐	☐	☐	☐	☐	

UNEMOTIONAL: Subject is relatively placid and unlikely to become aroused, upset, happy, or sad.

least								most
	1	2	3	4	5	6	7	
	☐	☐	☐	☐	☐	☐	☐	

IMITATIVE: Subject often mimics, or copies behaviors that it has observed in other chimpanzees.

least								most
	1	2	3	4	5	6	7	
	☐	☐	☐	☐	☐	☐	☐	

INDEPENDENT: Subject is individualistic and determines its own course of action without control or interference from other chimpanzees.

least								most
	1	2	3	4	5	6	7	
	☐	☐	☐	☐	☐	☐	☐	

UTAFITI WA TABIA (HULKA) YA SOKWE

Hulka ya sokwe inaweza kujulikana kwa kujaza alama mbalimbali kwa kila tabia kama inavyoelekezwa katika kurasa zinazofuata. Tumia uzoefu wako pamoja na maelezo yanayofuata kutoa alama kwa kila tabia ya sokwe. Masuala ya msingi ya kuzingatia wakati wa kujaza alama ni tabia ya sokwe akiwa peke yake na vilevile akijumuika na wenzake. Tumia maelezo yafuatayo hapa chini pamoja na uzoefu wako kuamua kama tabia inayoonyeshwa na sokwe ni ya kawaida, ni zaidi ya kawaida, au pungufu kuliko kawaida.

1. Haonyeshi tabia hii au ipo kwa kiasi kidogo sana.
2. Anaonyesha tabia mara chache sana.
3. Anaonyesha tabia kiasi kidogo kuliko kawaida.
4. Anaonyesha tabia hii kwa wastani/kawaida.
5. Anaonyesha tabia hii zaidi ya kawaida.
6. Anaonyesha tabia hii zaidi ya kawaida mara kwa mara.
7. Anaonyesha tabia hii kwa wingi.

Tafadhali jaza alama kwa kila tabia hata kama una wasiwasi na maamuzi yako. Weka alama hii ya ‘x’ chini ya kiboma ulichochagua. [X]

Mwisho, usijadiliane na mwenzako kuhusu ulivyotoa alama kwa sokwe yeyote. Kwa kufanya hivyo utasaidia kupata majibu na uhakika wa tabia nzima ya sokwe.

UTAFITI WA TABIA YA SOKWE

Jina kamili la sokwe: ____

Jina la Mtafiti: ____

Tarehe(Mwezi/Siku/Mwaka): ____

---

1. MWOGA: Kunapokuwa na hatari ya kweli au hatari ya kudhaniwa, huonyesha woga kupita kiasi kwa kupiga kelele, kukimbia au kuonyesha dalili za wasiwasi.

Kiasi kidogo sana **kiasi kikubwa sana**

1 ☐ 2 ☐ 3 ☐ 4 ☐ 5 ☐ 6 ☐ 7 ☐

---

2. MTAWALA: Huweza kufukuza, kutishia au kuchukua chakula kutoka kwa sokwe wengine. Au huweza kuonyesha utawala kwa kutuliza fujo ama kuvuruga amani ya wengine.

Kiasi kidogo sana **kiasi kikubwa sana**

1 ☐ 2 ☐ 3 ☐ 4 ☐ 5 ☐ 6 ☐ 7 ☐

---

3. MNG'ANG'ANIZI: Huwa na kawaida ya kuendelea na kitu au jambo au mbinu anayoifanya kwa muda mrefu licha ya kupata upinzani kutoka kwa sokwe wengine.

Kiasi kidogo sana **kiasi kikubwa sana**

1 ☐ 2 ☐ 3 ☐ 4 ☐ 5 ☐ 6 ☐ 7 ☐

---

4. MWANGALIFU: Huonekana kuwa makini kwa matendo yanayo weza kumzuru au kumletea hatari. Huepuka matendo yenye madhara.

Kiasi kidogo sana **kiasi kikubwa sana**

1 ☐ 2 ☐ 3 ☐ 4 ☐ 5 ☐ 6 ☐ 7 ☐

5. IMARA / MTULIVU: Huonyesha tabia kulingana na mazingira na sokwe wengine kwa utulivu, usawa na **mjio**. Tabia za sokwe wengine hazimkasirishi kirahisi.

Kiasi kidogo sana **kiasi kikubwa sana**

1	2	3	4	5	6	7
☐	☐	☐	☐	☐	☐	☐

---

6. UTOTO: Huendeleza na kurudia tabia fulani za kawaida kama vile kubembeleza na kujikumbatia.

Kiasi kidogo sana **kiasi kikubwa sana**

1	2	3	4	5	6	7
☐	☐	☐	☐	☐	☐	☐

---

7. MPEKUZI: Hupenda kujua juu ya sokwe wengine au kitu chochote akionacho. Pia hupenda kufuatilia mambo yasiyomhusu kuhusu sokwe wengine.

Kiasi kidogo sana **kiasi kikubwa sana**

1	2	3	4	5	6	7
☐	☐	☐	☐	☐	☐	☐

---

8. KUTOFIKIRIA: Anafanya matendo yake mara nyingi kizembe na kisahaulifu.

Kiasi kidogo sana **kiasi kikubwa sana**

1	2	3	4	5	6	7
☐	☐	☐	☐	☐	☐	☐

---

9. TAMAA: Huwa na tamaa ya chakula, sehemu nzuri au kitu chochote chenye thamani kwenye mazingira kupita kiasi. Hayuko tayari kuchangia chakula, sehemu nzuri au kitu chochote chenye thamani na wengine.

Kiasi kidogo sana **kiasi kikubwa sana**

1	2	3	4	5	6	7
☐	☐	☐	☐	☐	☐	☐

10. WIVU: Huwa na kawaida ya kuchukia kama wengine wako kwenye mazingira mazuri yanayoweza kuwapatia chakula, eneo zuri au kujenga urafiki na wengine. Au hujaribu kumvurugia mwenzake aliyeko katika nafasi nzuri ya kufanikiwa.

Kiasi kidogo sana **kiasi kikubwa sana**

1	2	3	4	5	6	7
☐	☐	☐	☐	☐	☐	☐

---

11. YAKIPEKEE: Matendo yake huonyesha kuwa ya kipekee. Hii haimaanishi kwamba hapatani na wengine.

Kiasi kidogo sana **kiasi kikubwa sana**

1	2	3	4	5	6	7
☐	☐	☐	☐	☐	☐	☐

---

12. MZEMBE: Hufanya matendo bila kujali kama yanaleta madhara kwake au kwa wengine.

Kiasi kidogo sana **kiasi kikubwa sana**

1	2	3	4	5	6	7
☐	☐	☐	☐	☐	☐	☐

---

13. MCHANGAMFU: Hupenda kujiunga na kuchanganyika na sokwe wengine kwa amani, furaha na urafiki.

Kiasi kidogo sana **kiasi kikubwa sana**

1	2	3	4	5	6	7
☐	☐	☐	☐	☐	☐	☐

---

14. MKURUPUSHAJI: Anaonekana kuwa rahisi kubughudhiwa na rahisi kukata tamaa mapema.

Kiasi kidogo sana **kiasi kikubwa sana**

1	2	3	4	5	6	7
☐	☐	☐	☐	☐	☐	☐

15. MWOGA: Hajiamini na hushtuka kirahisi na pia husita kushiriki kwenye mazingira mapya awe pekee au kwenye kikundi

Kiasi kidogo sana **kiasi kikubwa sana**

1	2	3	4	5	6	7
☐	☐	☐	☐	☐	☐	☐

---

16. MWENYE HURUMA: Huonyesha huruma na hisani kwa wengine na huonyesha kuguswa na matatizo yao na huwa tayari kuwatia moyo.

Kiasi kidogo sana **kiasi kikubwa sana**

1	2	3	4	5	6	7
☐	☐	☐	☐	☐	☐	☐

---

17. MCHANGAMFU: Hufurahia kucheza michezo ya kuchangamsha kama vile kuviringika, kuning'inia, mieleka au sarakasi akiwa peke yake au na wengine.

Kiasi kidogo sana **kiasi kikubwa sana**

1	2	3	4	5	6	7
☐	☐	☐	☐	☐	☐	☐

---

18. MPWEKE: Hupendelea kuwa peke yake na mara nyingi hapendi kujiunga na sokwe wengine.

Kiasi kidogo sana **kiasi kikubwa sana**

1	2	3	4	5	6	7
☐	☐	☐	☐	☐	☐	☐

---

19. ROHO NYEPESI: Rahisi kuumia kimwili au kiakili kutokana na kujijenga, kushambuliwa, au tabia zinazotokana na utawala na ujeuri wa sokwe wengine.

Kiasi kidogo sana **kiasi kikubwa sana**

1	2	3	4	5	6	7
☐	☐	☐	☐	☐	☐	☐

20. MBUNIFU: Huonyesha tabia mpya au mambo mapya kama vile katika kucheza na wenzake au kutumia vitu.

Kiasi kidogo sana **kiasi kikubwa sana**

1	2	3	4	5	6	7
☐	☐	☐	☐	☐	☐	☐

---

21. MTENDAJI: Sio mzembe au mvivu na anaonekana kupendelea kutumia muda wake mwingi kuzungukazunguka au kujihusisha na vitendo vinavyotumia nguvu.

Kiasi kidogo sana **kiasi kikubwa sana**

1	2	3	4	5	6	7
☐	☐	☐	☐	☐	☐	☐

---

22. MHISANI (MOYO MZURI): Huwa tayari kusaidiana au kushirikiana na wenzake.

Kiasi kidogo sana **kiasi kikubwa sana**

1	2	3	4	5	6	7
☐	☐	☐	☐	☐	☐	☐

---

23. MUONEVU: Hutishia na kuonea sokwe wadogo na wale wenye hadhi ndogo.

Kiasi kidogo sana **kiasi kikubwa sana**

1	2	3	4	5	6	7
☐	☐	☐	☐	☐	☐	☐

---

24. MJEURI: Mara nyingi huanzisha fujo au mapigano au matishio mengine yenye kuleta fujo na ushindani.

Kiasi kidogo sana **kiasi kikubwa sana**

1	2	3	4	5	6	7
☐	☐	☐	☐	☐	☐	☐

25. MWEREVU: Mtaalamu wa kujiunga kwenye vikundi kwa manufaa yake pekee hususani, hupenda kuwatumia wengine au kutumia urafiki ili kupanda kiutawala. Huonyesha nia na uwezo wa kutumia wenzake.

Kiasi kidogo sana **kiasi kikubwa sana**

1	2	3	4	5	6	7
☐	☐	☐	☐	☐	☐	☐

---

26. MPOLE: Huonyesha upole, wema na utulivu kwa wenzake. Haonyeshi ukorofi au vitisho kwa wengine.

Kiasi kidogo sana **kiasi kikubwa sana**

1	2	3	4	5	6	7
☐	☐	☐	☐	☐	☐	☐

---

27. UPENDO: Huonyesha mapenzi na ukaribu kwa sokwe wengine kwa kukwatuana, kukumbatiana, kugusana au kupumzika (kulala) karibu na wengine.

Kiasi kidogo sana **kiasi kikubwa sana**

1	2	3	4	5	6	7
☐	☐	☐	☐	☐	☐	☐

---

28. MACHACHARI: Husisimshwa kirahisi na hali ambayo kawaida haisisimshi sokwe wengi kirahisi.

Kiasi kidogo sana **kiasi kikubwa sana**

1	2	3	4	5	6	7
☐	☐	☐	☐	☐	☐	☐

---

29. HAELEWEKI: Anafanya mambo ghafla bila kutegemewa ambayo yanaonyesha kusukumwa na hisia zake.

Kiasi kidogo sana **kiasi kikubwa sana**

1	2	3	4	5	6	7
☐	☐	☐	☐	☐	☐	☐

30. MDADISI: Huvutiwa na hali mpya, vitu vipya au wanyama wapya. Huonekana kutaka kujifunza kuhusu sokwe wengine, vitu au watu walio karibu.

Kiasi kidogo sana **kiasi kikubwa sana**

1 ☐ 2 ☐ 3 ☐ 4 ☐ 5 ☐ 6 ☐ 7 ☐

---

31. MTIIFU: Mara nyingi hunyenyekea na kutii sokwe wengine. Anafanya matendo kama vile yeye ndie mdogo kiutawala kuliko sokwe wengine.

Kiasi kidogo sana **kiasi kikubwa sana**

1 ☐ 2 ☐ 3 ☐ 4 ☐ 5 ☐ 6 ☐ 7 ☐

---

32. MTULIVU: Huwa mtulivu wa moyo na mara nyingi huwa mwenye amani na uhakika.

Kiasi kidogo sana **kiasi kikubwa sana**

1 ☐ 2 ☐ 3 ☐ 4 ☐ 5 ☐ 6 ☐ 7 ☐

---

33. MTEGEMEA/ MFUATAJI: Mara nyingi hutegemea kupata muongozo na matumaini kutoka kwa sokwe wengine na pia hutafuta kukumbatiana au kushikana na sokwe wengine.

Kiasi kidogo sana **kiasi kikubwa sana**

1 ☐ 2 ☐ 3 ☐ 4 ☐ 5 ☐ 6 ☐ 7 ☐

---

34. MTUKUTU: Huonekana kununa au kutokuwa na subira na hughadhibika kirahisi na kuanzisha ugomvi.

Kiasi kidogo sana **kiasi kikubwa sana**

1 ☐ 2 ☐ 3 ☐ 4 ☐ 5 ☐ 6 ☐ 7 ☐

35. MJINGA: Si mwepesi wa kuelewa hali, au tabia za wengine.

Kiasi kidogo sana **kiasi kikubwa sana**

1 ☐ 2 ☐ 3 ☐ 4 ☐ 5 ☐ 6 ☐ 7 ☐

---

36. TABIRIKA: Huwa na tabia isiyobadilika na huendeleza tabia hii kwa muda mrefu. Huonyesha tabia isiyotabirika au hubadili tabia yake ya kawaida kwa nadra sana.

Kiasi kidogo sana **kiasi kikubwa sana**

1 ☐ 2 ☐ 3 ☐ 4 ☐ 5 ☐ 6 ☐ 7 ☐

---

37. MWAMUA: Huwa makini kwenye nyendo zake.

Kiasi kidogo sana **kiasi kikubwa sana**

1 ☐ 2 ☐ 3 ☐ 4 ☐ 5 ☐ 6 ☐ 7 ☐

---

38. SONONEKA: Hapendi kujiunga na wengine na hushindwa kushirikiana na sokwe wengine. Mara nyingi huwa mbali, pekee, hununa na kuhuzunika na hupunguza matendo. Huonekana mkiwa.

Kiasi kidogo sana **kiasi kikubwa sana**

1 ☐ 2 ☐ 3 ☐ 4 ☐ 5 ☐ 6 ☐ 7 ☐

---

39. MFUATA DESTURI: Hukosa ujanja wa kuanzisha tabia mpya au tofauti. Huonyesha tabia zile zile daima bila kubadili na hufuata tabia zinazokubalika katika jamii yake.

Kiasi kidogo sana **kiasi kikubwa sana**

1 ☐ 2 ☐ 3 ☐ 4 ☐ 5 ☐ 6 ☐ 7 ☐

40. MWEPESI KUHISI: Huweza kuhisi na kuelewa hali, silika, mawazo au dhamira za sokwe wengine hata kutokana na ishara zisizotambulika kirahisi.

Kiasi kidogo sana **kiasi kikubwa sana**

1	2	3	4	5	6	7
☐	☐	☐	☐	☐	☐	☐

---

41. MKAIDI: Huonyesha kujiamini na huleta ushindani bila kujali wanao mzidi kiutawala. Huendeleza tabia hizi bila matokeo mabaya au kujali matishio au mashambulio toka kwa sokwe wengine.

Kiasi kidogo sana **kiasi kikubwa sana**

1	2	3	4	5	6	7
☐	☐	☐	☐	☐	☐	☐

---

42. MWELEVU: Mwepesi wa kutabiri na kuelewa mambo yote barabara, yawe ni ya kijamii au yasiyo ya kijamii. Hufahamu na hutambua mahusiano ya kijamii.

Kiasi kidogo sana **kiasi kikubwa sana**

1	2	3	4	5	6	7
☐	☐	☐	☐	☐	☐	☐

---

43. MLINZI: Hujali hali ya sokwe wengine na mara nyingi huingilia kati ili kuepusha madhara au maudhi kwa sokwe wengine.

Kiasi kidogo sana **kiasi kikubwa sana**

1	2	3	4	5	6	7
☐	☐	☐	☐	☐	☐	☐

---

44. ACHA KITENDO: Huanzisha jambo na kuliacha katikati bila kusita.

Kiasi kidogo sana **kiasi kikubwa sana**

1	2	3	4	5	6	7
☐	☐	☐	☐	☐	☐	☐

45. MTENGENEZAJI: Ana uwezo zaidi ya wengine wa kujaribu mambo mapya au kuonyesha tabia mpya. Tabia mpya ni pamoja na njia mpya ya kutumia vitu kwenye mazingira yake.

Kiasi kidogo sana **kiasi kikubwa sana**

1 ☐ 2 ☐ 3 ☐ 4 ☐ 5 ☐ 6 ☐ 7 ☐

---

46. YUMBA: Hana utaratibu au mwelekeo kwa mfano wakati wakutembea au michezo au mambo mengine.

Kiasi kidogo sana **kiasi kikubwa sana**

1 ☐ 2 ☐ 3 ☐ 4 ☐ 5 ☐ 6 ☐ 7 ☐

---

47. BADILIKABADILIKA: Hubadili tabia na hali mara kwa mara na tabia moja hutofautiana sana na nyingine.

Kiasi kidogo sana **kiasi kikubwa sana**

1 ☐ 2 ☐ 3 ☐ 4 ☐ 5 ☐ 6 ☐ 7 ☐

---

48. URAFIKI: Hupendelea kuungana na sokwe wengine kwenye shughuli za kirafiki na za kuchangamsha. Huanzisha ugomvi na sokwe wengine kwa nadra sana.

Kiasi kidogo sana **kiasi kikubwa sana**

1 ☐ 2 ☐ 3 ☐ 4 ☐ 5 ☐ 6 ☐ 7 ☐

---

49. PAPARIKA: Huonyesha dhiki, wasiwasi, fadhaa au mashaka.

Kiasi kidogo sana **kiasi kikubwa sana**

1 ☐ 2 ☐ 3 ☐ 4 ☐ 5 ☐ 6 ☐ 7 ☐

50. MVIVU: Anaonekana kuwa goigoi au mlegevu na hupenda kutembea taratibu au kujiepusha na michezo ya nguvu.

**Kiasi kidogo sana**						**kiasi kikubwa sana**
1	2	3	4	5	6	7
☐	☐	☐	☐	☐	☐	☐

---

51. HANA MPANGILIO: Huwa sio makini katika matendo yake. Hukosa mpangilio katika nyendo au tabia zake kana kwamba hana mwelekeo.

**Kiasi kidogo sana**						**kiasi kikubwa sana**
1	2	3	4	5	6	7
☐	☐	☐	☐	☐	☐	☐

---

52. KUTOGUSWA (KUTOJARI): Huwa mara nyingi amepoa na haonyeshi hisia yeyote. Haelekei kushtushwa, kufadhaishwa au kuhuzunishwa wala kufurahishwa.

**Kiasi kidogo sana**						**kiasi kikubwa sana**
1	2	3	4	5	6	7
☐	☐	☐	☐	☐	☐	☐

---

53. IGIZA: Mara nyingi ana uwezo wa kuigiza vitendo alivyoona kwa sokwe mwingine.

**Kiasi kidogo sana**						**kiasi kikubwa sana**
1	2	3	4	5	6	7
☐	☐	☐	☐	☐	☐	☐

---

54. JITEGEMEA: Hujitegemea kipekee na hufanya maamuzi yake pekee bila kuongozwa au kuingiliwa na sokwe wengine.

**Kiasi kidogo sana**						**kiasi kikubwa sana**
1	2	3	4	5	6	7
☐	☐	☐	☐	☐	☐	☐

UTAFITI WA TABIA (HULKA) YA SOKWE

Hulka ya sokwe inaweza kujulikana kwa kujaza alama mbalimbali kwa kila tabia kama inavyoelekezwa katika kurasa zinazofuata. Tumia uzoefu wako pamoja na maelezo yanayofuata kutoa alama kwa kila tabia ya sokwe. Masuala ya msingi ya kuzingatia wakati wa kujaza alama ni tabia ya sokwe akiwa peke yake na vilevile akijumuika na wenzake. Tumia maelezo yafuatayo hapa chini pamoja na uzoefu wako kuamua kama tabia inayoonyeshwa na sokwe ni ya kawaida, ni zaidi ya kawaida, au pungufu kuliko kawaida.

1. Haonyeshi tabia hii au ipo kwa kiasi kidogo sana.
2. Anaonyesha tabia mara chache sana.
3. Anaonyesha tabia kiasi kidogo kuliko kawaida.
4. Anaonyesha tabia hii kwa wastani/kawaida.
5. Anaonyesha tabia hii zaidi ya kawaida.
6. Anaonyesha tabia hii zaidi ya kawaida mara kwa mara.
7. Anaonyesha tabia hii kwa wingi.

Tafadhali jaza alama kwa kila tabia hata kama una wasiwasi na maamuzi yako. Weka alama hii ya ‘x’ chini ya kiboma ulichochagua. [X]

Mwisho, usijadiliane na mwenzako kuhusu ulivyotoa alama kwa sokwe yeyote. Kwa kufanya hivyo utasaidia kupata majibu na uhakika wa tabia nzima ya sokwe.

UTAFITI WA TABIA YA SOKWE

Jina kamili la sokwe: ____

Jina la Mtafiti: ____

Tarehe(Mwezi/Siku/Mwaka): _______________________________________

---

1. MTAWALA: Huweza kufukuza, kutishia au kuchukua chakula kutoka kwa sokwe wengine. Au huweza kuonyesha utawala kwa kutuliza fujo ama kuvuruga amani ya wengine.

Kiasi kidogo sana **kiasi kikubwa sana**

1	2	3	4	5	6	7
☐	☐	☐	☐	☐	☐	☐

---

2. MPWEKE: Hupendelea kuwa peke yake na mara nyingi hapendi kujiunga na sokwe wengine.

Kiasi kidogo sana **kiasi kikubwa sana**

1	2	3	4	5	6	7
☐	☐	☐	☐	☐	☐	☐

---

3. HAELEWEKI: Anafanya mambo ghafla bila kutegemewa ambayo yanaonyesha kusukumwa na hisia zake.

Kiasi kidogo sana **kiasi kikubwa sana**

1	2	3	4	5	6	7
☐	☐	☐	☐	☐	☐	☐

---

4. MWENYE HURUMA: Huonyesha huruma na hisani kwa wengine na huonyesha kuguswa na matatizo yao na huwa tayari kuwatia moyo.

Kiasi kidogo sana **kiasi kikubwa sana**

1	2	3	4	5	6	7
☐	☐	☐	☐	☐	☐	☐

5. IMARA / MTULIVU: Huonyesha tabia kulingana na mazingira na sokwe wengine kwa utulivu, usawa na mjio. Tabia za sokwe wengine hazimkasirishi kirahisi.

Kiasi kidogo sana **kiasi kikubwa sana**

1 ☐ 2 ☐ 3 ☐ 4 ☐ 5 ☐ 6 ☐ 7 ☐

---

6. MTENGENEZAJI: Ana uwezo zaidi ya wengine wa kujaribu mambo mapya au kuonyesha tabia mpya. Tabia mpya ni pamoja na njia mpya ya kutumia vitu kwenye mazingira yake.

Kiasi kidogo sana **kiasi kikubwa sana**

1 ☐ 2 ☐ 3 ☐ 4 ☐ 5 ☐ 6 ☐ 7 ☐

---

7. MTEGEMEA/ MFUATAJI: Mara nyingi hutegemea kupata muongozo na matumaini kutoka kwa sokwe wengine na pia hutafuta kukumbatiana au kushikana na sokwe wengine.

Kiasi kidogo sana **kiasi kikubwa sana**

1 ☐ 2 ☐ 3 ☐ 4 ☐ 5 ☐ 6 ☐ 7 ☐

---

8. MCHANGAMFU: Hupenda kujiunga na kuchanganyika na sokwe wengine kwa amani, furaha na urafiki.

Kiasi kidogo sana **kiasi kikubwa sana**

1 ☐ 2 ☐ 3 ☐ 4 ☐ 5 ☐ 6 ☐ 7 ☐

---

9. KUTOFIKIRIA: Anafanya matendo yake mara nyingi kizembe na kisahaulifu.

Kiasi kidogo sana **kiasi kikubwa sana**

1 ☐ 2 ☐ 3 ☐ 4 ☐ 5 ☐ 6 ☐ 7 ☐

10. MHISANI (MOYO MZURI): Huwa tayari kusaidiana au kushirikiana na wenzake.

Kiasi kidogo sana **kiasi kikubwa sana**

1 ☐ 2 ☐ 3 ☐ 4 ☐ 5 ☐ 6 ☐ 7 ☐

---

11. MACHACHARI: Husisimshwa kirahisi na hali ambayo kawaida haisisimshi sokwe wengi kirahisi.

Kiasi kidogo sana **kiasi kikubwa sana**

1 ☐ 2 ☐ 3 ☐ 4 ☐ 5 ☐ 6 ☐ 7 ☐

---

12. MDADISI: Huvutiwa na hali mpya, vitu vipya au wanyama wapya. Huonekana kutaka kujifunza kuhusu sokwe wengine, vitu au watu walio karibu.

Kiasi kidogo sana **kiasi kikubwa sana**

1 ☐ 2 ☐ 3 ☐ 4 ☐ 5 ☐ 6 ☐ 7 ☐

---

13. MWAMUA: Huwa makini kwenye nyendo zake.

Kiasi kidogo sana **kiasi kikubwa sana**

1 ☐ 2 ☐ 3 ☐ 4 ☐ 5 ☐ 6 ☐ 7 ☐

---

14. YAKIPEKEE: Matendo yake huonyesha kuwa ya kipekee. Hii haimaanishi kwamba hapatani na wengine.

Kiasi kidogo sana **kiasi kikubwa sana**

1 ☐ 2 ☐ 3 ☐ 4 ☐ 5 ☐ 6 ☐ 7 ☐

15. MZEMBE: Hufanya matendo bila kujali kama yanaleta madhara kwake au kwa wengine.

Kiasi kidogo sana **kiasi kikubwa sana**

1 2 3 4 5 6 7

☐ ☐ ☐ ☐ ☐ ☐ ☐

---

16. MWEPESI KUHISI: Huweza kuhisi na kuelewa hali, silika, mawazo au dhamira za sokwe wengine hata kutokana na ishara zisizotambulika kirahisi.

Kiasi kidogo sana **kiasi kikubwa sana**

1 2 3 4 5 6 7

☐ ☐ ☐ ☐ ☐ ☐ ☐

---

17. KUTOGUSWA (KUTOJARI): Huwa mara nyingi amepoa na haonyeshi hisia yeyote. Haelekei kushtushwa, kufadhaishwa au kuhuzunishwa wala kufurahishwa.

Kiasi kidogo sana **kiasi kikubwa sana**

1 2 3 4 5 6 7

☐ ☐ ☐ ☐ ☐ ☐ ☐

---

18. MPEKUZI: Hupenda kujua juu ya sokwe wengine au kitu chochote akionacho. Pia hupenda kufuatilia mambo yasiyomhusu kuhusu sokwe wengine.

Kiasi kidogo sana **kiasi kikubwa sana**

1 2 3 4 5 6 7

☐ ☐ ☐ ☐ ☐ ☐ ☐

---

19. ROHO NYEPESI: Rahisi kuumia kimwili au kiakili kutokana na kujijenga, kushambuliwa, au tabia zinazotokana na utawala na ujeuri wa sokwe wengine.

Kiasi kidogo sana **kiasi kikubwa sana**

1 2 3 4 5 6 7

☐ ☐ ☐ ☐ ☐ ☐ ☐

20. MTENDAJI: Sio mzembe au mvivu na anaonekana kupendelea kutumia muda wake mwingi kuzungukazunguka au kujihusisha na vitendo vinavyotumia nguvu.

Kiasi kidogo sana **kiasi kikubwa sana**

1	2	3	4	5	6	7
☐	☐	☐	☐	☐	☐	☐

---

21. TABIRIKA: Huwa na tabia isiyobadilika na huendeleza tabia hii kwa muda mrefu. Huonyesha tabia isiyotabirika au hubadili tabia yake ya kawaida kwa nadra sana.

Kiasi kidogo sana **kiasi kikubwa sana**

1	2	3	4	5	6	7
☐	☐	☐	☐	☐	☐	☐

---

22. MFUATA DESTURI: Hukosa ujanja wa kuanzisha tabia mpya au tofauti. Huonyesha tabia zile zile daima bila kubadili na hufuata tabia zinazokubalika katika jamii yake.

Kiasi kidogo sana **kiasi kikubwa sana**

1	2	3	4	5	6	7
☐	☐	☐	☐	☐	☐	☐

---

23. MTULIVU: Huwa mtulivu wa moyo na mara nyingi huwa mwenye amani na uhakika.

Kiasi kidogo sana **kiasi kikubwa sana**

1	2	3	4	5	6	7
☐	☐	☐	☐	☐	☐	☐

---

24. MBUNIFU: Huonyesha tabia mpya au mambo mapya kama vile katika kucheza na wenzake au kutumia vitu.

Kiasi kidogo sana **kiasi kikubwa sana**

1	2	3	4	5	6	7
☐	☐	☐	☐	☐	☐	☐

Chimp ID	Chimp Birth Year	Sex	H (1969-2005)	X (1971-2005)	G (1972-2005)	P (1977-2005)	F (1980-2009)	J (1985)	O (1987-2007)	Y (1991)	U (1991)	V (1996)	C (1999-2009)	B (1999)	Ratings per chimp
Z329	1919	F	X	X	X										3
P186	1944	M	X	X	X										3
G333	1947	M	X	X	X										3
P302	1937	M	X	X	X										3
G103	1936	M	X	X	X										3
A412	1945	F	X	X	X										3
E163	1938	M	X	X	X										3
P80	1949	M	X	X	X										3
R475	1951	M	X	X	X										3
T466	1946	M	X	X	X										3
U376	1953	M	X	X	X										3
C154	1949	F	X	X	X										3
D38	1956	F	X	X	X										3
U228	1952	M	X	X	X										3
H340	1954	F	X	X	X										3
D152	1958	F	X	X	X										3
Y440	1959	M	X	X	X										3
C332	1949	F	X	X	X										3
Q315	1956	M	X	X	X										3
F17	1948	M	X	X	X										3
C141	1953	M	X		X										2
X489	1953	F	X	X	X										3
R312	1952	F	X	X	X										3
B89	1952	F	X	X	X										3
H389	1950	F	X	X	X										3
I142	1955	M	X	X	X										3
M203	1961	M	X	X	X										3
W10	1960	F		X	X	X									3
G334	1965	F		X	X	X									3
I355	1965	F			X	X	X								3
U150	1969	F			X	X	X								3
K205	1961	F		X	X	X									3
L22	1960	F				X	X	X							3
M171	1964	M				X	X	X							3
N309	1965	M				X	X	X							3
G19	1967	M				X	X	X							3
Q257	1958	F				X	X	X							3
Q461	1970	F				X	X	X							3
J295	1970	F				X	X	X							3
O468	1957	F				X	X	X							3
W399	1971	M				X	X	X							3
Y57	1961	F				X	X	X							3
D191	1971	M				X	X	X			X				4
S250	1958	F				X	X	X							3
T215	1973	M				X	X	X							3
N319	1969	M				X	X	X							3
K405	1976	M				X			X	X	X		X		5
G195	1977	F			X	X	X								3
H275	1978	F			X		X								2
Y342	1971	M				X	X	X							3
W148	1941	F	X		X										2
Q96	1969	F				X	X								2
K398	1966	F				X	X	X							3
M26	1972	F					X	X	X						3
J452	1978	F					X	X	X						3
M162	1976	M					X	X	X						3
X261	1969	F				X	X	X							3
J448	1984	M					X	X	X						3
V128	1985	M					X	X	X						3
K262	1972	M							X	X	X				3
E131	1973	F							X						1
Z189	1973	F							X	X	X				3
T362	1977	M							X	X	X				3
N161	1977	M							X	X	X				3
J234	1977	M							X	X	X		X		4
P70	1979	M							X	X	X				3
W432	1981	F							X	X	X				3
B24	1982	M							X	X	X				3
L210	1983	M							X	X	X				3
P325	1984	M							X	X	X				3
Q450	1984	F							X	X	X				3
Y235	1985	F							X	X	X				3
V52	1973	F							X	X	X				3
D138	1978	F							X	X	X				3
D224	1981	F							X	X	X				3
W359	1970	F				X	X	X							3
M63	1993	F								X		X	X	X	4
B396	1986	F										X	X	X	3
A383	1988	M										X	X	X	3
U464	1989	M										X		X	2
L112	1989	M										X	X	X	3
Q72	1989	F										X	X	X	3
V467	1991	F										X	X	X	3
M395	1991	F										X	X	X	3
M193	1992	M										X	X	X	3
F145	1993	M										X	X	X	3
I415	1994	M										X	X	X	3
N478	1996	M										X	X	X	3
K480	1996	M										X	X	X	3
Z201	1988	F										X	X	X	3
J83	1983	F										X	X	X	3
V331	1984	F										X	X	X	3
F357	1985	F										X	X	X	3
U97	1998	F										X	X	X	3
C133	1998	F										X	X	X	3
K206	1998	F										X	X	X	3
P109	1998	F										X	X	X	3
F225	1999	M										X	X	X	3
E125	1999	F										X	X	X	3
K497	2000	M										X	X	X	3
R81	1988	F										X	X	X	3
Q277	1978	F							X	X	X				3
A100	1989	F										X	X	X	3
R439	1990	F										X	X	X	3
Q217	1972	F							X	X	X				3
J278	1992	F										X	X	X	3
C352	1992	F										X	X	X	3
			28	29	35	26	28	23	24	19	19	29	30	29	319
														Min	1
														Max	5
														Med	3

Min	Max	Med
19	35	28

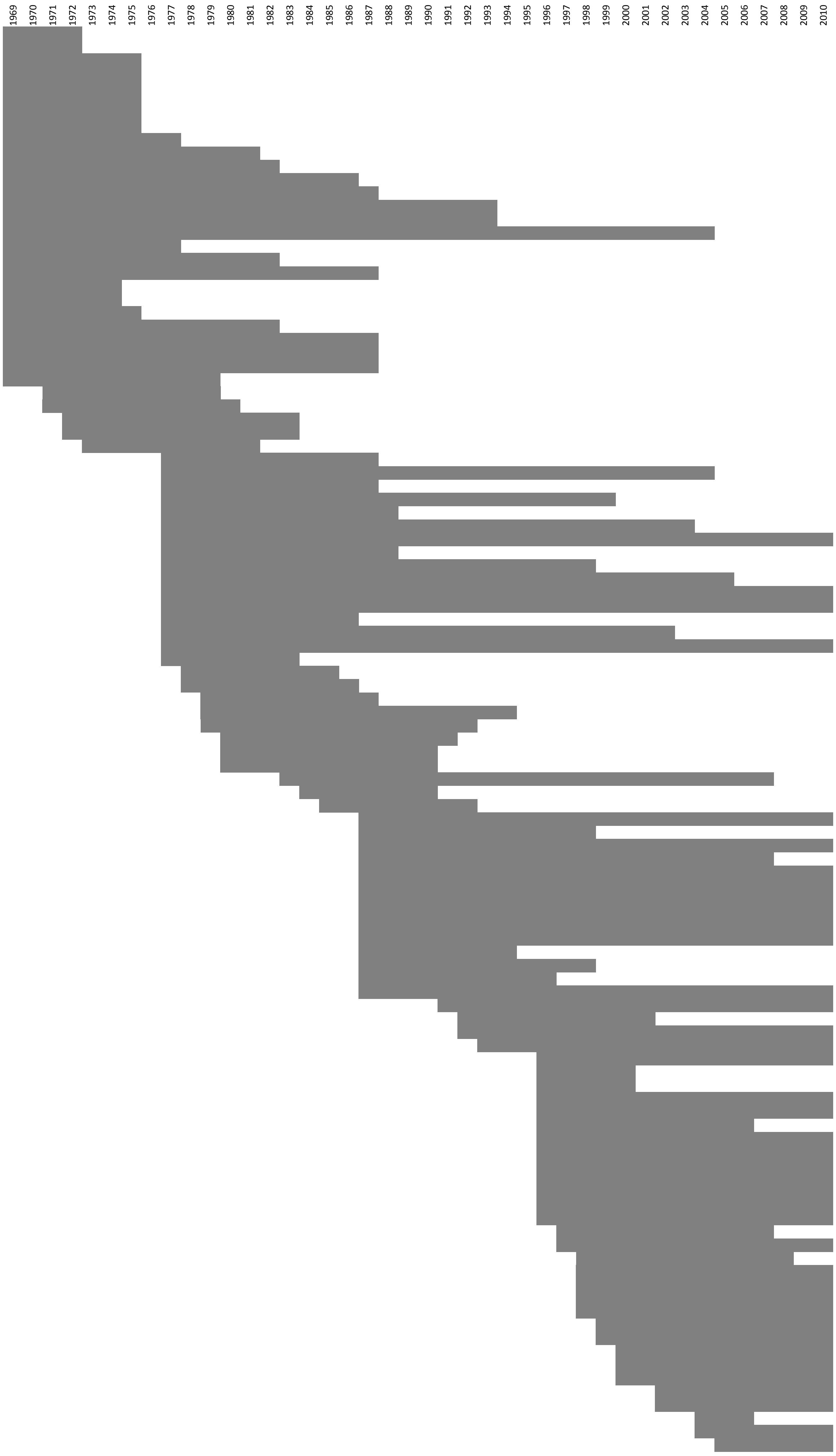

Note. Raters without an end date were still employed as field assistants when they made their ratings. Chimpanzees rated on the EPI have shaded ID cells.

																															Rater (Employment Dates)									
Chimp ID	Chimp birth year	Sex	First seen	Last seen	1986	1987	1988	1989	1990	1991	1992	1993	1994	1995	1996	1997	1998	1999	2000	2001	2002	2003	2004	2005	2006	2007	2008	2009	2010	2011	Q (1985-2007)	N (1988)	A (1997)	E (2002)	W (2002)	M (2003)	Ratings per chimp			
A341	1986	M	1986	2011																											X	X	X	X		X	5			
E131	1973	F	1988	2011																											X	X	X	X	X	X	6			
A364	1992	F	1992	2003																											X	X	X				3			
K227	1980	F	1993	2011																											X	X	X	X	X	X	6			
N108	1988	F	1993	2005																											X	X	X	X	X	X	6			
K135	1976	M	1993	2004																											X	X	X	X	X	X	6			
K58	1992	F	1994	2011																											X	X	X	X	X	X	6			
X213	1984	F	1994	2011																											X	X	X	X	X	X	6			
N251	1965	F	1995	2010																											X	X	X	X	X	X	6			
M37	1989	M	1995	2011																											X	X	X	X	X	X	6			
Y235	1985	F	1996	2011																											X	X	X	X	X	X	6			
E274	1996	M	1996	2005																											X	X	X	X	X	X	6			
E421	1997	M	1997	2011																											X	X	X	X	X	X	6			
O450	1984	F	1998	2011																											X	X	X	X	X	X	6			
G74	1998	M	1998	2011																											X	X	X	X	X	X	6			
O454	2000	M	2001	2011																											X	X	X	X	X	X	6			
E182	1986	F	2001	2011																											X	X	X	X	X	X	6			
K199	1997	F	2001	2009																											X	X	X	X	X	X	6			
N101	2001	M	2001	2011																											X	X	X	X	X	X	6			
O198	2001	M	2001	2011																											X	X	X	X	X	X	6			
W394	2001	M	2002	2011																											X	X	X	X	X	X	6			
Y207	2004	F	2004	2011																											X	X	X	X	X	X	6			
J499	1991	F	2004	2011																											X	X	X	X	X	X	6			
T137	2005	F	2005	2011																											X	X	X	X	X	X	6	min	max	median
																															24	24	24	23	22	23	140	22	24	23.5
																																				min	3			
																																				max	6			
																																				med	6			

Note. Raters without an end date were still employed as field assistants when they made their ratings.

Table S3: Varimax rotation of six factor solution

Item	Factor							Item's loading in previous studies*
	I	II	III	IV	V	VI	h^2	
Dominant	**0.79**	0.13	0.17	0.25	0.11	-0.04	0.74	Dominance (+)
Reckless	**0.65**	0.14	0.34	0.02	0.08	-0.15	0.59	Conscientiousness (-)
Excitable	**0.60**	0.15	0.12	0.27	**0.41**	-0.06	0.63	Neuroticism (+)
Impulsive	**0.55**	-0.03	**0.40**	0.11	0.24	-0.13	0.56	Conscientiousness (-)
Inventive	**0.51**	0.02	0.05	0.32	0.26	0.11	0.44	Openness (+)
Sociable	0.10	**0.76**	-0.02	0.09	0.29	0.01	0.67	Extraversion (+)
Dependent/follower	0.00	**0.61**	0.07	-0.14	-0.07	0.12	0.42	Dominance (-)
Innovative	**0.44**	**0.50**	-0.17	-0.11	0.29	0.10	0.58	Openness (+)
Solitary	-0.12	**-0.49**	0.23	-0.22	-0.25	0.08	0.43	Extraversion (-)
Active	0.07	0.11	**0.64**	**0.45**	0.07	-0.16	0.66	Extraversion (+)
Predictable	0.14	-0.01	**0.60**	0.11	0.06	0.10	0.41	Conscientiousness (+)
Individualistic	0.18	-0.13	**0.55**	-0.08	0.00	0.03	0.35	Extraversion (-)
Sensitive	0.30	-0.24	0.01	**0.67**	0.29	0.24	0.73	Agreeableness (+)
Decisive	0.19	0.05	0.12	**0.60**	0.05	0.10	0.42	Dominance (+)
Inquisitive	0.36	0.28	-0.01	0.16	**0.71**	0.06	0.75	Openness (+)
Curious	0.27	0.22	0.29	0.16	**0.56**	0.10	0.56	Openness (+)
Helpful	0.34	**0.47**	0.16	0.16	0.11	**0.60**	0.76	Agreeableness (+)
Sympathetic	-0.17	-0.03	-0.10	0.03	-0.01	**0.59**	0.39	Agreeableness (+)
Stable	-0.02	0.12	0.22	0.25	0.10	0.38	0.28	Neuroticism (-)
Proportion of variance	0.14	0.10	0.09	0.08	0.08	0.06		

Note. h^2 = Proportion of variance in each item accounted for by all six factors.
*Direction of the association between each item and chimpanzee personality dimensions identified in previous studies.

Table S4: Varimax rotation of four factor solution

Item	Factor I	II	III	IV	h^2	Item's loading in previous studies*
Sensitive	**0.82**	-0.13	0.05	0.29	0.77	Agreeableness (+)
Excitable	**0.64**	0.36	0.26	-0.20	0.65	Neuroticism (+)
Inventive	**0.60**	0.20	0.17	-0.01	0.43	Openness (+)
Dominant	**0.57**	0.30	0.37	-0.23	0.61	Dominance (+)
Inquisitive	**0.55**	**0.53**	0.06	-0.04	0.59	Openness (+)
Decisive	**0.47**	0.03	0.18	0.23	0.31	Dominance (+)
Curious	**0.43**	0.39	0.31	0.05	0.44	Openness (+)
Sociable	0.09	**0.76**	0.01	0.08	0.59	Extraversion (+)
Innovative	0.22	**0.69**	-0.06	-0.12	0.54	Openness (+)
Dependent/follower	-0.26	**0.56**	0.08	0.13	0.40	Dominance (-)
Helpful	0.26	**0.55**	0.23	**0.42**	0.60	Agreeableness (+)
Solitary	-0.24	**-0.51**	0.18	0.01	0.35	Extraversion (-)
Predictable	0.11	-0.01	**0.64**	0.13	0.44	Conscientiousness (+)
Individualistic	0.02	-0.10	**0.56**	-0.03	0.32	Extraversion (-)
Active	0.27	0.01	**0.56**	0.07	0.39	Extraversion (+)
Impulsive	**0.44**	0.13	**0.53**	-0.26	0.56	Conscientiousness (-)
Reckless	0.33	0.28	**0.50**	-0.34	0.55	Conscientiousness (-)
Sympathetic	-0.03	0.00	-0.13	**0.50**	0.27	Agreeableness (+)
Stable	0.16	0.12	0.21	**0.48**	0.31	Neuroticism (-)
Proportion of variance	0.17	0.14	0.11	0.06		

Note. h^2 = Proportion of variance in each item accounted for by all four factors.
*Direction of the association between each item and chimpanzee personality dimensions identified in previous studies.

Table S5: Promax rotation of six component solution

Item	Component I	II	III	IV	V	VI	h^2	Item's loading in previous studies*
Dominant	**0.87**	-0.06	0.15	0.07	-0.20	0.01	0.70	Dominance (+)
Excitable	**0.85**	-0.01	-0.02	0.23	-0.29	0.04	0.75	Neuroticism (+)
Reckless	**0.75**	0.02	0.36	-0.03	-0.30	-0.06	0.67	Conscientiousness (-)
Inventive	**0.69**	-0.18	-0.04	-0.06	0.26	-0.02	0.62	Openness (+)
Impulsive	**0.68**	-0.13	0.38	-0.04	0.04	-0.20	0.65	Conscientiousness (-)
Inquisitive	**0.64**	0.24	-0.17	0.01	0.08	-0.04	0.64	Openness (+)
Innovative	**0.59**	0.43	-0.17	-0.37	-0.04	0.07	0.70	Openness (+)
Curious	**0.45**	0.23	0.14	0.11	0.21	-0.05	0.51	Openness (+)
Sensitive	**0.43**	-0.39	-0.14	0.37	0.31	0.10	0.76	Agreeableness (+)
Dependent/follower	-0.21	**0.82**	0.16	-0.04	-0.03	0.12	0.66	Dominance (-)
Sociable	0.03	**0.81**	-0.12	0.01	0.24	-0.19	0.76	Extraversion (+)
Solitary	-0.14	**-0.53**	**0.44**	-0.25	0.06	0.18	0.61	Extraversion (-)
Individualistic	0.13	-0.10	**0.75**	-0.20	0.28	-0.08	0.62	Extraversion (-)
Predictable	0.07	0.06	**0.68**	0.35	0.01	0.15	0.63	Conscientiousness (+)
Decisive	0.16	-0.07	-0.06	**0.76**	-0.04	0.15	0.68	Dominance (+)
Active	-0.04	0.15	**0.47**	**0.63**	0.11	-0.25	0.75	Extraversion (+)
Stable	-0.29	0.21	0.25	0.02	**0.95**	0.05	0.79	Neuroticism (-)
Sympathetic	-0.08	-0.09	-0.04	0.05	0.04	**0.90**	0.83	Agreeableness (+)
Helpful	0.31	0.39	0.16	0.04	0.19	**0.49**	0.73	Agreeableness (+)
Proportion of variance	0.23	0.12	0.10	0.09	0.07	0.07		

Component Correlations

I	II	III	IV	V	
0.31					II
0.02	-0.03				III
0.34	0.04	0.04			IV
0.40	0.03	-0.15	0.29		V
-0.04	0.12	-0.09	-0.01	0.16	VI

Note. h^2 = Proportion of variance in each item accounted for by all six components.
*Direction of the association between each item and chimpanzee personality dimensions identified in previous studies.

Table S6: Varimax rotation of six component solution

Item	Component							Item's loading in previous studies*
	I	II	III	IV	V	VI	h^2	
Dominant	**0.79**	0.07	0.20	0.16	-0.01	-0.08	0.70	Dominance (+)
Excitable	**0.78**	0.13	0.05	0.31	0.02	-0.14	0.75	Neuroticism (+)
Inventive	**0.70**	-0.07	-0.02	0.08	0.01	0.34	0.62	Openness (+)
Inquisitive	**0.68**	0.34	-0.14	0.12	0.00	0.17	0.64	Openness (+)
Impulsive	**0.65**	-0.05	**0.41**	0.07	-0.22	0.07	0.65	Conscientiousness (-)
Reckless	**0.65**	0.12	**0.41**	0.04	-0.10	-0.23	0.67	Conscientiousness (-)
Innovative	**0.56**	**0.53**	-0.15	-0.28	0.10	0.01	0.70	Openness (+)
Curious	**0.55**	0.29	0.16	0.20	-0.01	0.24	0.51	Openness (+)
Sensitive	**0.50**	-0.30	-0.13	**0.46**	0.13	**0.42**	0.76	Agreeableness (+)
Sociable	0.24	**0.79**	-0.12	0.05	-0.11	0.22	0.76	Extraversion (+)
Dependent/follower	-0.10	**0.77**	0.15	-0.07	0.14	-0.09	0.66	Dominance (-)
Solitary	-0.26	**-0.54**	**0.41**	-0.25	0.13	-0.01	0.61	Extraversion (-)
Individualistic	0.14	-0.12	**0.73**	-0.13	-0.09	0.18	0.62	Extraversion (-)
Predictable	0.12	0.06	**0.69**	0.36	0.12	0.00	0.63	Conscientiousness (+)
Decisive	0.26	-0.02	-0.03	**0.76**	0.15	0.09	0.68	Dominance (+)
Active	0.14	0.11	**0.49**	**0.63**	-0.25	0.11	0.75	Extraversion (+)
Sympathetic	-0.14	-0.03	-0.07	0.03	**0.89**	0.10	0.83	Agreeableness (+)
Helpful	0.39	**0.47**	0.15	0.11	**0.52**	0.23	0.73	Agreeableness (+)
Stable	0.01	0.15	0.19	0.12	0.15	**0.83**	0.79	Neuroticism (-)
Proportion of variance	0.23	0.13	0.10	0.09	0.07	0.07		

Note. h^2 = Proportion of variance in each item accounted for by all six components.
*Direction of the association between each item and chimpanzee personality dimensions identified in previous studies.

Table S7: Promax rotation of four component solution

Item	Component I	II	III	IV	h^2	Item's loading in previous studies[*]
Sensitive	**0.98**	-0.32	-0.11	0.31	0.75	Agreeableness (+)
Inventive	**0.73**	0.02	-0.02	0.02	0.52	Openness (+)
Decisive	**0.68**	-0.15	0.04	0.33	0.46	Dominance (+)
Excitable	**0.64**	0.19	0.06	-0.17	0.69	Neuroticism (+)
Dominant	**0.54**	0.14	0.21	-0.21	0.66	Dominance (+)
Inquisitive	**0.53**	**0.46**	-0.14	-0.01	0.64	Openness (+)
Curious	0.37	0.33	0.21	0.09	0.50	Openness (+)
Sociable	-0.09	**0.88**	-0.07	0.11	0.66	Extraversion (+)
Dependent/follower	**-0.60**	**0.84**	0.19	0.16	0.63	Dominance (-)
Innovative	0.08	**0.76**	-0.19	-0.10	0.62	Openness (+)
Helpful	0.16	**0.59**	0.17	**0.49**	0.67	Agreeableness (+)
Solitary	-0.23	**-0.59**	0.39	0.01	0.48	Extraversion (-)
Individualistic	-0.18	-0.16	**0.79**	-0.04	0.53	Extraversion (-)
Predictable	-0.04	-0.06	**0.78**	0.19	0.57	Conscientiousness (+)
Active	0.19	-0.10	**0.64**	0.09	0.50	Extraversion (+)
Impulsive	0.38	-0.04	**0.46**	-0.27	0.62	Conscientiousness (-)
Reckless	0.20	0.17	**0.44**	-0.36	0.62	Conscientiousness (-)
Sympathetic	0.07	0.07	-0.14	**0.72**	0.53	Agreeableness (+)
Stable	0.15	0.14	0.27	**0.66**	0.52	Neuroticism (-)
Proportion of variance	0.20	0.17	0.13	0.09		

Component correlations I	II	III	
0.46			II
0.43	0.26		III
-0.18	-0.17	-0.13	IV

Note. h^2 = Proportion of variance in each item accounted for by all four components.
[*]Direction of the association between each item and chimpanzee personality dimensions identified in previous studies.

Table S8: Varimax rotation of four component solution

Item	Component I	II	III	IV	h^2	Item's loading in previous studies*
Sensitive	**0.80**	-0.14	0.03	0.29	0.75	Agreeableness (+)
Excitable	**0.69**	0.35	0.22	-0.19	0.69	Neuroticism (+)
Inventive	**0.69**	0.18	0.12	0.01	0.52	Openness (+)
Dominant	**0.63**	0.30	0.35	-0.23	0.66	Dominance (+)
Decisive	**0.58**	-0.03	0.13	0.32	0.46	Dominance (+)
Inquisitive	**0.58**	**0.55**	0.03	-0.03	0.64	Openness (+)
Curious	**0.47**	**0.42**	0.31	0.07	0.50	Openness (+)
Sociable	0.08	**0.81**	0.00	0.08	0.66	Extraversion (+)
Innovative	0.22	**0.75**	-0.08	-0.13	0.62	Openness (+)
Dependent/follower	-0.36	**0.68**	0.15	0.14	0.63	Dominance (-)
Solitary	-0.27	**-0.58**	0.27	0.03	0.48	Extraversion (-)
Helpful	0.26	**0.58**	0.22	**0.46**	0.67	Agreeableness (+)
Predictable	0.11	-0.01	**0.73**	0.18	0.57	Conscientiousness (+)
Individualistic	-0.02	-0.11	**0.72**	-0.04	0.53	Extraversion (-)
Active	0.29	0.00	**0.64**	0.08	0.50	Extraversion (+)
Impulsive	**0.49**	0.12	**0.53**	-0.28	0.62	Conscientiousness (-)
Reckless	0.37	0.29	**0.51**	-0.37	0.62	Conscientiousness (-)
Sympathetic	-0.04	-0.01	-0.17	**0.71**	0.53	Agreeableness (+)
Stable	0.15	0.12	0.26	**0.64**	0.52	Neuroticism (-)
Proportion of variance	0.20	0.17	0.13	0.09		

Note. h^2 = Proportion of variance in each item accounted for by all four components.
*Direction of the association between each item and chimpanzee personality dimensions identified in previous studies.

Table S9: Interrater reliabilities of chimpanzees in the Kasekela community separated by whether the rater was employed as a field assistant when the chimpanzee was rated and whether the chimpanzee was alive when it was rated

	Not working[a]	Working[b]	Deceased[c]	Living[d]
Dominance	0.27	0.22	0.23	0.27
Extraversion	0.16	0.44	0.13	0.16
Conscientiousness	0.34	0.04	0.30	0.08
Agreeableness	0.23	0.20	0.14	0.24
Neuroticism	0.11	-0.04	0.17	0.15
Openness	0.38	0.13	0.30	0.24

Note. Analyses restricted to the Kasekela community as only one rater of the Mitumba community chimpanzees was no longer employed and only three chimpanzees in the Mitumba community were deceased. [a]Reliability estimates based on 97 ratings of 34 chimpanzees by 4 raters. [b]Reliability estimates based on 97 ratings of 47 chimpanzees by 5 raters. [c]Reliability estimates based on 166 ratings of 57 chimpanzees by 12 raters. [d]Reliability estimates based on 152 ratings of 49 chimpanzees by 11 raters.
